# TinyOdom: Hardware-Aware Efficient Neural Inertial Navigation

**DOI:** 10.1145/3534594

**Published:** 2022-07-07

**Authors:** SWAPNIL SAYAN SAHA, SANDEEP SINGH SANDHA, LUIS ANTONIO GARCIA, MANI SRIVASTAVA

**Affiliations:** University of California - Los Angeles, USA; University of California - Los Angeles, USA; University of Southern California, USA; University of California - Los Angeles, USA

**Keywords:** inertial odometry, dead-reckoning, sequence-learning, resource-constrained devices, neural architecture search, hardware-in-the-loop, machine-learning, deep-learning, tracking

## Abstract

Deep inertial sequence learning has shown promising odometric resolution over model-based approaches for trajectory estimation in GPS-denied environments. However, existing neural inertial dead-reckoning frameworks are not suitable for real-time deployment on ultra-resource-constrained (URC) devices due to substantial memory, power, and compute bounds. Current deep inertial odometry techniques also suffer from gravity pollution, high-frequency inertial disturbances, varying sensor orientation, heading rate singularity, and failure in altitude estimation. In this paper, we introduce TinyOdom, a framework for training and deploying neural inertial models on URC hardware. TinyOdom exploits hardware and quantization-aware Bayesian neural architecture search (NAS) and a temporal convolutional network (TCN) backbone to train lightweight models targetted towards URC devices. In addition, we propose a magnetometer, physics, and velocity-centric sequence learning formulation robust to preceding inertial perturbations. We also expand 2D sequence learning to 3D using a model-free barometric g-h filter robust to inertial and environmental variations. We evaluate TinyOdom for a wide spectrum of inertial odometry applications and target hardware against competing methods. Specifically, we consider four applications: pedestrian, animal, aerial, and underwater vehicle dead-reckoning. Across different applications, TinyOdom reduces the size of neural inertial models by 31× to 134× with 2.5m to 12m error in 60 seconds, enabling the direct deployment of models on URC devices while still maintaining or exceeding the localization resolution over the state-of-the-art. The proposed barometric filter tracks altitude within ±0.1*m* and is robust to inertial disturbances and ambient dynamics. Finally, our ablation study shows that the introduced magnetometer, physics, and velocity-centric sequence learning formulation significantly improve localization performance even with notably lightweight models.

## 1 INTRODUCTION

Odometry is the fusion of onboard sensors for indirect estimation of an object’s position and attitude under absence or in conjunction with infrastructure-dependent localization services [5]. Given the widespread ubiquity of inertial measurement units (IMU), inertial odometry [18, 44, 46, 53, 77] is a viable alternative available to localization applications demanding small footprint, low-access delay, low-power pathway, and operating in GPS or network-denied environments. Examples of such applications include terrestrial and marine “search and rescue” missions [82], underwater sensor networks [15], oceanic biodiversity and marine health tracking [88], wildlife monitoring [89], deep-space small satellite localization [73], and localizing micro unmanned vehicles and robots [10, 29, 96]. For example, marine search and rescue missions [82] limit the compute device payload and resource availability (e.g., rescuers can only carry limited weight), and cannot assume continuous access to GPS or network infrastructure (e.g., the rescue operation can happen underground). The use-case of marine health tracking [88] and wildlife monitoring [89] necessitate odometry solutions that can operate in the absence of infrastructure and have a light payload of deployment hardware not impacting the normal animal behavior. Further, localizing micro unmanned vehicles and robots [10, 29, 96] demand a small footprint solution due to compute and energy constraints. These environments represent scenarios where compute devices are ultra-resource constrained, thus necessitating the need to develop lightweight approaches where the smartphone or cloud-based solutions are unusable. However, adopting internal odometry for resource-constrained hardware and across different use-cases is challenging, as discussed next.

### 1.1 Challenges

To handle the error explosion inherent in inertial navigation, inertial odometry on URC microcontroller-class hardware is heavily hard-coded through application-specific heuristics, Bayesian statistics, and human-engineered system models. These techniques, although lightweight, are not robust to domain shifts or inertial perturbations [10, 16, 46]. Recently, several data-driven techniques based on sequence learning have been used in the attempt to alleviate the constraints in model-based approaches for various applications [10, 29, 35, 74, 96]. However, enabling accurate yet lightweight and real-time neural-inertial methods for resource-constrained environments faces the following challenges

- **The Real-time Deployment on URC devices:** While neural-inertial methods have been shown to provide superior long-term resolution over classical techniques [16, 46, 74], they are unsuitable for real-time deployment [18, 35] on URC hardware due to memory, power, and compute constraints. Consequently, no prior work has shown neural-inertial architectures running in real-time under extremely resource-constrained settings (e.g., 128 kB RAM, 1 MB flash).
- **The Run-Time Robustness of Existing Approaches:** Existing neural-inertial methods suffer from gravity pollution, high-frequency inertial artifacts, varying device attitude, heading-rate singularity, and 3D estimation failure. The current solutions come at the cost of larger neural network models, auxiliary ML operations, or the addition of model-based filters [29, 46, 59, 74, 96].
- **The Realization of 3-D Odometry:** While classical methods can perform 3D odometry via sensor fusion without significant compute overhead, the data-driven neural-inertial methods thus far have been mostly limited to 2D tracking only [59]. Data-driven methods which attempt to perform 3D tracking with inertial sensors suffer from the curse of inertial drift and gravity pollution.

### 1.2 Contributions

We introduce TinyOdom, a systematic and practical framework for deploying lightweight yet robust 3D inertial odometry models on URC hardware. TinyOdom leverages advances in NAS to optimize inertial sequence learning models based on hardware constraints, accuracy, and latency goals via direct communication with target hardware. In addition, TinyOdom uses a magnetometer, physics, and velocity-centric sequence learning formulation with a TCN backbone, allowing tiny models to perform accurate inference even under inertial disturbances while maintaining the simplicity of models. To expand 2D tracking to 3D, we perform sensor fusion using barometric g-h filters robust to inertial and environmental variations. To showcase the generalizability of TinyOdom, we evaluate pedestrian, animal, aerial, and underwater vehicle dead-reckoning on four different URC hardware platforms. TinyOdom reduces the neural network model size between 31× to 134× with 2.5m to 12m error in 60 seconds over the state-of-the-art (SOTA), thereby enabling the direct deployment of neural-inertial odometry using the onboard compute resources of URC hardware platforms. Even though our neural network models are notably lightweight, the introduced magnetometer, physics, and velocity-centric sequence learning formulation still maintain or exceed the tracking performance compared to the existing state-of-the-art. We evaluate the proposed barometric g-h filter showing it outperforms the baselines and is robust to pressure sensor noises in real data. The superior and lightweight real-time inertial tracking enabled by TinyOdom holds the key to improving the tracking performance of applications deployed in challenging GPS/network denied environments. Our work not only enables always-on and lightweight neural-inertial navigation, but also improves the cost and the energy footprint of embedded odometry while being expandable to any reduced footprint hardware.

Our contributions are summarized as follows:

- We propose a magnetometer, physics, and velocity-centric inertial sequence formulation to generate models robust to gravity pollution, high-frequency inertial perturbations, varying sensor attitude, and heading rate singularity, without adding significant compute overhead.
- We develop a hardware-in-the-loop (HIL) AutoML framework based on Bayesian Optimization (BO) to generate lightweight inertial odometry models without sacrificing resolution significantly. We use a TCN backbone as the basis for NAS and TensorFlow Lite Micro (TFLM) as model runtime interpreter.
- We exploit the omnipresence of barometers to expand 2D dead-reckoning to 3D using lightweight barometric *α – β* filters that are robust to inertial and environmental variations. The filter can perform altitude tracking within ±0.1m.
- We extensively benchmark TinyOdom for pedestrian, animal, aerial, and underwater vehicle dead-reckoning on four different URC hardware platforms against competing inertial odometry baselines, based on accuracy and resource usage.
- To the best of our knowledge, we are the first to showcase a real-world evaluation of neural-inertial navigation and discuss challenges and solutions of transferring pre-trained odometry models in the real world.

TinyOdom is available open-source^1^ to promote development and benchmarking of lightweight yet robust neural inertial odometry that is generalizable across different applications.

### 1.3 Organization

The rest of the paper is organized as follows: Section 2 mathematically motivates the challenges of localization using an inertial sensor. Section 3 presents the related work that attempts to mitigate the challenges in inertial tracking, as well as recent advances in lightweight deep-learning. Section 4 details the robust 3D inertial sequence learning formulation. Section 5 delineates the model architecture and the hardware-aware NAS formulation. Section 6 presents the experimental setup, baseline algorithms, and datasets used for evaluation. Section 7 presents extensive experimental evaluation of the models generated by the TinyOdom framework. Finally, Section 8 provides concluding remarks and future directions.

## 2 BACKGROUND

MEMS inertial sensors are usually equipped with a 3DoF accelerometer, a 3DoF gyroscope, and a 3DoF magnetometer [50]. *Firstly*, when the gyroscope is mounted on an immobile platform w.r.t Earth frame close to the Earth’s surface, the gyroscope within a MEMS inertial sensor is modeled as follows [50]:

(1) $$\omega_{ib}=\omega_{nb}+b_{g}+n_{g}$$

where, ${\dot{b}}_{g}∼𝓝\left(0,Q_{g}\right)=\text{ bias gradient }$, $n_{g}∼𝓝\left(0,{\text{Σ}}_{\omega }\right)=\text{ additive white Gaussian noise (AWGN) }$, and *ω*_*nb*_ = latent and uncorrupted angular velocity. *Secondly*, assuming negligible effects centrifugal or Coriolis components of Earth’s rotation, the accelerometer model is defined as [50]:

(2) $$f^{b}=R^{bn}\left(a_{nn}^{n}-g\right)+b_{a}+n_{a}$$

where, *a*_𝑛𝑛_ = latent linear acceleration of body, g=gravity vector , ${\dot{b}}_{a}∼𝓝\left(0,Q_{a}\right)=\text{ bias gradient }$, and $n_{a}∼𝓝\left(0,{\text{Σ}}_{a}\right)=\text{AWGN}$. *Lastly*, the compass can be modeled as [50]:

(3) $$b^{n}=R^{bn}m^{n}+n_{b},\;m^{n}=\left[\begin{array}{ccc} cos\delta & 0 & sin\delta \end{array}\right]$$

where, $n_{b}∼𝓝\left(0,{\text{Σ}}_{m}\right)=\text{AWGN}$, and *δ* = magnetic inclination due to Earth’s magnetic dip. Under ideal geomagnetic conditions void of magnetic disturbances, non-uniform magnetic field, or sensor noise, and for slow movements, the heading *H* can be estimated directly from the magnetometer [88] in the body frame *I* :

(4) $$H=\arctan\left(\frac{m_{y,t}^{I}}{-m_{x,t}^{I}}\right)\cdot \frac{180}{\pi }$$

The latitude (*ϕ*) and longitude (*λ*) can be hypothetically obtained via double integration of accelerometer readings ($a_{x}^{I}$, $a_{y}^{I}$, $a_{z}^{I}$) under empirical accelerometer vector sum threshold *α* [88] to reject noise:

(5) $$s_{t}=\left\{\begin{array}{ll} \beta \sqrt{{\;}^{I}a_{x,t}^{2}{+}^{I}a_{y,t}^{2}{+}^{I}a_{z,t}^{2}}+\gamma , & \sqrt{{\;}^{I}a_{x,t}^{2}{+}^{I}a_{y,t}^{2}{+}^{I}a_{z,t}^{2}}>\alpha \\ 0 & \text{otherwise} \end{array}\right.$$

(6) $$\phi_{t}=\arcsin\left(\sin\phi_{t-1}\cdot \cos\frac{s_{t}}{R_{E}}+\cos\phi_{t-1}\cdot \sin\frac{s_{t}}{R_{E}}\cdot \cos H\right),\;R_{E}=6.371\times 10^{6}\text{m}$$

(7) $$\lambda_{t}=\lambda_{t-1}+\arctan2\left(\sin H\cdot \sin\frac{s_{t}}{R_{E}}\cdot \cos\phi_{t-1},\cos\frac{s_{t}}{R_{E}}-\sin\phi_{t-1}\cdot \sin\phi_{t}\right)$$

**Fig. 1.**
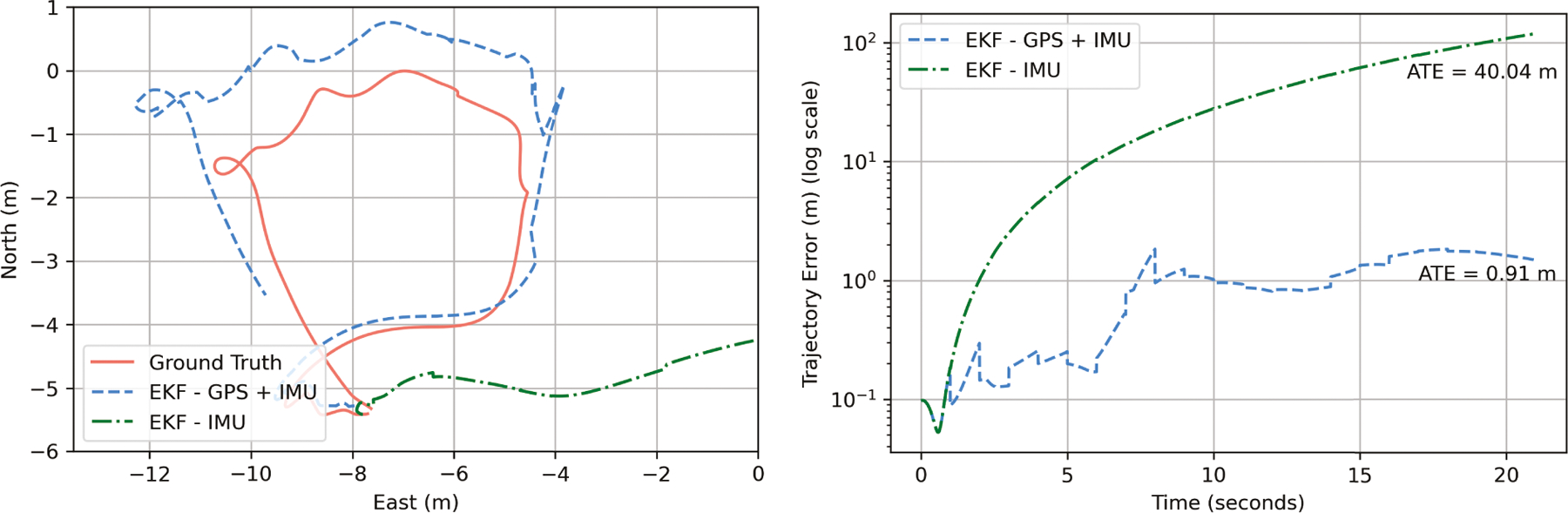
Example of localization of a quadrotor using GPS+IMU and autonomous IMU via Extended Kalman Filter (EKF). (Left) GPS corrects drift induced by naive double integration of accelerometer readings, while using autonomous IMU without error correction heuristics leads to cumulative drift that explodes with time. (Right) Error in trajectory estimation grows cubically with time when autonomous IMU is used without error correction, while GPS constrains the error within a few meters. ATE refers to absolute trajectory error.

While magnetometers are unpolluted by device motion [70], magnetic disturbances coupled with sensor placement offset can affect the seemingly simple estimation of *H* and lead to errors as much as 100° [94]. Furthermore, naive double integration (NDI) of accelerometer readings cumulatively accumulates the effects of time-varying bias (*b*_*a*_), gravity pollution (*g*), and AWGN (*n*_*a*_), causing errors in *ϕ* and *λ* to explode in a cubic manner [46, 62, 70, 93], illustrated in Fig. 1. The gyroscope suffers from time-varying drift (*b*_*g*_) in the long term because of bias instability (BI) and angular random walk (ARW) resulting from AWGN (*n*_*g*_), pink noise and thermal effects [62]. The cubic explosion of error in position estimate, *σ*_*x*_ (*t*), due to gyroscope drift can be modelled as:

(8) $$\sigma_{x}(t)=v\sqrt{{\left(2\cdot ARW\cdot \sqrt{t^{3}}/3\right)}^{2}+\left(BI\cdot t^{2}/2\right)}$$

The goal of inertial odometry is to address the cumulative error in naive double integration of accelerometer readings. Conventional approaches use model-based methods that are dependent on application-specific heuristics and demand expert domain knowledge. More recently, learning-enabled approaches are proposed that either work in combination with model-based methods or work in an end-to-end manner, completely replacing model-based methods. In the next section, we present the existing state-of-the-art in both model-based methods and learning-enabled approaches. We also briefly discuss recent advances in lightweight deep learning.

## 3 RELATED WORK

In this section, we provide a comprehensive review of the recent advances in inertial odometry and efficient DL for URC devices, outlining their strengths and weaknesses. Inertial localization can be categorized into model-based systems using Bayesian filters and heuristics, or learning-enabled systems exploiting recent advances in DL [46]. Software advances in TinyML include the use of pruning, quantization and model compression, lightweight neural blocks, hardware-aware NAS and the rise of commercial off-the-shelf (COTS) tools [64, 67]

### 3.1 Model-Based Inertial Odometry

Model-based inertial dead-reckoning frameworks typically employ multimodal fusion via physics-based and heuristic priors with occasional support from infrastructure-dependent position fixes, along with the application of Bayesian filters for error handling [46]. These approaches are commonly hard-coded for specific applications.

#### 3.1.1 Vehicular, Robotic and Animal Localization.

Depending on the target application and topography, heuristic drift reduction, magnetic anomaly detection, opportunistic calibration, quasi-static moment detection, particle filters, magnetic map matching, and inertial signature verification are used to counteract heading estimation error [47, 70, 91, 94, 99], typically fused through an error-tracking indirect Kalman filter (KF). For displacement estimation, unmanned aerial, ground, and underwater vehicles (UAV, UGV, and UUV) typically fuse inertial sensors with GPS, LIDAR, camera, and RADAR via KF variants for localization, complemented via map information from known localization space [3, 53]. Typical aid includes magnetic map matching, flow-sensors, and wheel odometers, with nonholonomic constraints on the motion. Satellites and spacecraft perform non-linear Bayesian fusion of physics-based kinematics models with inertial sensor measurements for attitude estimation [22], coupled with position information from GPS, kinematics models (e.g. ephemeris and almanac), relative angle measurements (e.g. parallax) or ground control [38, 61]. For slow and predictable motion profiles such as in wildlife tracking, ecologists formulate animal-specific belief-based constraints on velocity, transportation modes, and Boolean decision-trees to mitigate drifts and sensor errors, occasionally fusing GPS through KF variants when available [87–89].

#### 3.1.2 Pedestrian Dead Reckoning (PDR).

PDR systems typically decompose position estimation problems into direction estimation and stride length estimation [94], the latter of which is further partitioned into transportation mode classification, gait cycle segmentation, and step length estimation (SLE) subject to environmental constraints and iterative updates [44, 47, 91]. For dead-reckoning via foot-mounted IMU, information about the linear and angular velocities of the foot during the swing and stance phases for various motion primitives can be exploited to constrain the explosion of errors in step counting [33, 47, 91]. This is referred to as zero velocity update (ZUPT) or zero angular rate update (ZARU) pseudo-measurements [49], segregating the gait cycle into identifiable chunks either coarsely or granularly [47]. Temporal and frequency-domain analysis (e.g., wavelet and Fourier transforms, level crossing detection, extrema detection, autocorrelation, Hidden Markov Models (HMM), and local variance detection) are used to extract recurrent and temporal contextual dynamics in the gait cycle to improve the robustness of gait phase identification against AWGN for various activity modes [2, 47, 91, 94]. Typical SLE approaches include physiological knowledge injection (e.g., Weinberg SLE), linear regression upon step frequency with aiding covariance heuristics, global acceleration extrema difference, knowledge of virtual landmarks, and floor plans or use of KF variants [44, 47, 56, 83, 91, 94].

While model-based inertial localization systems are computationally efficient, designing generalizable heuristics poses a significant hurdle in the deployment of real-time inertial dead-reckoning systems, with no “one size fits all” analytical solution to the problem. The system models used by model-based approaches are linear approximations of the state evolution in the real world, which do not translate accurately in the long run for eclectic scenarios due to non-optimal parametrization. In contrast, in TinyOdom, we propose an end-to-end learning-based framework that is generalizable across different applications. We show that TinyOdom develops machine learning models that are superior to model-based methods and are deployable on resource constraint devices. We outline existing data-driven techniques in the next subsection.

### 3.2 Data-Driven Inertial Odometry

To handle the shortcoming of model-based methods, researchers have recently proposed several ML approaches capable of capturing high-dimensional contextual dynamics in the non-linear domain void of human knowledge. Next, we categorize existing approaches based on the role of data-driven components. They are either used in combination with model-based methods or are completely replacing them in an end-to-end manner.

#### 3.2.1 Aiding Model-Based Systems.

Deep neural networks (DNN) are adept at filtering out noise and irrelevant information while extracting useful features from sensors in the wild [66]. Intuitively, a hands-off generalizable solution for the curse of drift in inertial odometry involves eliminating the root of sensing uncertainty on the fly before the model-based position estimation step using DL models. Affixing a neural network block dedicated to denoising in the Bayesian state estimation framework yields real-time and on-the-fly noise reduction while being invariant to random gyrations and actuator micro-vibrations, exhibiting generalizability under unseen trajectory projections and robustness to training data anomalies and domain shift [1, 11, 12, 98]. An alternative approach involves using DNN or reinforcement-learning (RL) agents to dynamically and tightly update KF noise covariance parameters instead of sensor errors [10, 36, 90]. In this case, the filter is non-agnostic to the corrections being made by the DNN, leading to estimates that are statistically Pareto-optimal under non-ideal gradient descent convergence. A parallel technique uses DNN as tightly coupled state advisors, providing indirect momentary pseudo-measurements about position and orientation (such as velocity and heading) from a finite window of raw inertial readings to physics-based filters to aid decision making under a broad spectrum of motion primitives, topography, environment, sensor-placement and test subjects [20, 59, 72].

#### 3.2.2 Velocity-Profile Heuristics.

For non-Bayesian filters such as in PDR, DL-based velocity profile detectors (ZUPT and ZARU) and SLE can extract coveted velocity intervals for step detection and adapt stride length based upon discerned motion patterns [80, 81] for varying gait patterns and sensor placements. In the case of wheeled robots, DL-based ZUPT and ZARU allow attitude and bias corrections, with priors on lateral and vertical velocity to improve long-term position estimation accuracy [9]. RIDI [93] used classical ML to detect sensor placement and regress velocity and direction to correct accelerometer readings for usable double integration within a stabilized-inertial frame for various transportation modes.

#### 3.2.3 End-to-End Frameworks.

Model-based filters require an accurate representation of the evolution of sensor errors and state estimates in terms of the incoming measurements. Such system models are only linear approximations and are unable to optimally control error explosion from naive double integration due to deviations from mathematical abstractions for non-linear complex motions or deployment in different domains [16, 46]. IONet [16] introduced the concept of sequence learning for dead-reckoning, resulting in the first end-to-end neural-inertial model capable of trajectory estimation in presence of non-linearities associated with inertial localization which are otherwise hard to model mathematically, including effects of unrestricted sensor orientation and position, different test subjects, diverse motion primitives and abnormalities, decoupling individual sensor errors and biases, sampling rate jitter and physical characteristics of the sensors. Notable successors of IONet include RoNIN [46], IDOL [74] and L-IONet [18] for PDR, AbolDeepIO for aerial vehicle localization [29], VeTorch for UGV localization [35] and NavNet [96] for UUV positioning. MotionTransformer uses a transformer network to generate domain invariant inertial sequences from raw sensor data from various sensor placements, rotations, or motion types in a completely unsupervised fashion, without requiring the exhaustive collection of labeled domain-specific datasets [17].

Out of the proposed data-driven methods, *data-driven end-to-end frameworks* are preferred, as they are not dependent on any domain heuristics and have shown superior performance [16, 18, 46] over model-based techniques. However, currently, none of the data-driven methods are suitable for real-time deployment on URC devices. Among all of the data-driven techniques, only L-IONet [18], VeTorch [35] and the two frameworks by Brossard et al. [10, 12] were designed with efficiency in mind to run in real-time on smartphones. The constraints of URC devices (e.g., a typical microcontroller has 128 KB RAM and 1 MB of flash) demand extremely lightweight machine learning models in comparison to a smartphone, which can have 4 GB of RAM and 64 GB of storage [57]. TinyOdom is designed to address the gap of enabling neural inertial odometry on ultra-resource-constrained devices. TinyOdom reduces the model size between 31× to 134× in comparison to the existing data-driven methods. Even though our models are lightweight, TinyOdom either maintains or exceeds the tracking performance in comparison to the existing SOTA due to a novel magnetometer, physics, and velocity-centric inertial sequence formulation.

### 3.3 Deep-Learning for URC devices

Several libraries exist that enable the transfer of trained machine learning models generated by well-known libraries (such as Tensorflow) to microcontrollers. These libraries include TensorFlow Lite Micro [23, 84], CMSIS-NN [54], uTensor [75], and Microsoft EdgeML [24, 25, 39, 40, 42, 51, 52, 65]. Such libraries provide comprehensive sets of optimized ML operators, algorithms, and tools, perform pruning, quantization (fixed and mixed precision), and model compression [43] and convert models to deployable C code. However, these libraries assume that the trained model can fit within the device resource constraints. To satisfy the tighter hardware constraints of URC devices, neural architecture search (NAS) needs to be optimized by target hardware specifications to strike a balance between accuracy and efficiency [30, 57] tradeoff.

Several NAS frameworks have been proposed for microcontroller-class devices. SpArSe [30] treats NAS as a gradient-driven multi-objective BO problem, treating hardware attributes via proxies and coupling pruning with NAS. MicroNets [6] uses a quantization-aware gradient-driven approach to optimize task-aware DNN backbones. MCUNet [57] tailors Once-for-All (OFA) NAS [14] for microcontrollers, using a two-stage evolutionary NAS to train a single OFA network in an optimized search space for a broad spectrum of target hardware. Adopting MCUNet is a challenge as it uses a custom inference engine and its latency/resource measurements rely on a closed-source software stack. In TinyOdom, we perform hardware-aware NAS using multi-objective BO, where the acquisition function is optimized using Monte Carlo sampling. We adopt BO due to the following reasons: (i) BO provides a state-of-the-art approach to optimize expensive objective functions in a few evaluations [69], (ii) BO allows explicit inclusion of non-gradient-friendly constraints of the model size and accuracy tradeoffs during the training process [30]. The choice of Monte Carlo sampling instead of the gradient-driven approach of SpArSe [30] is based on the fact that neural architecture search space consists of categorical variables where the sampling approach evaluates the acquisition function only at valid configurations only [37, 68]. TinyOdom includes the hardware-aware training where the resource utilization of a model is computed at runtime by its real deployment on the target hardware, instead of just using proxies as done by SpArSe [30]. Our evaluation shows that proxies are only approximations of the real hardware constraints, which are noisy for extremely resource-constrained devices.

## 4 ROBUST 3D INERTIAL SEQUENCE LEARNING

**Fig. 2.**
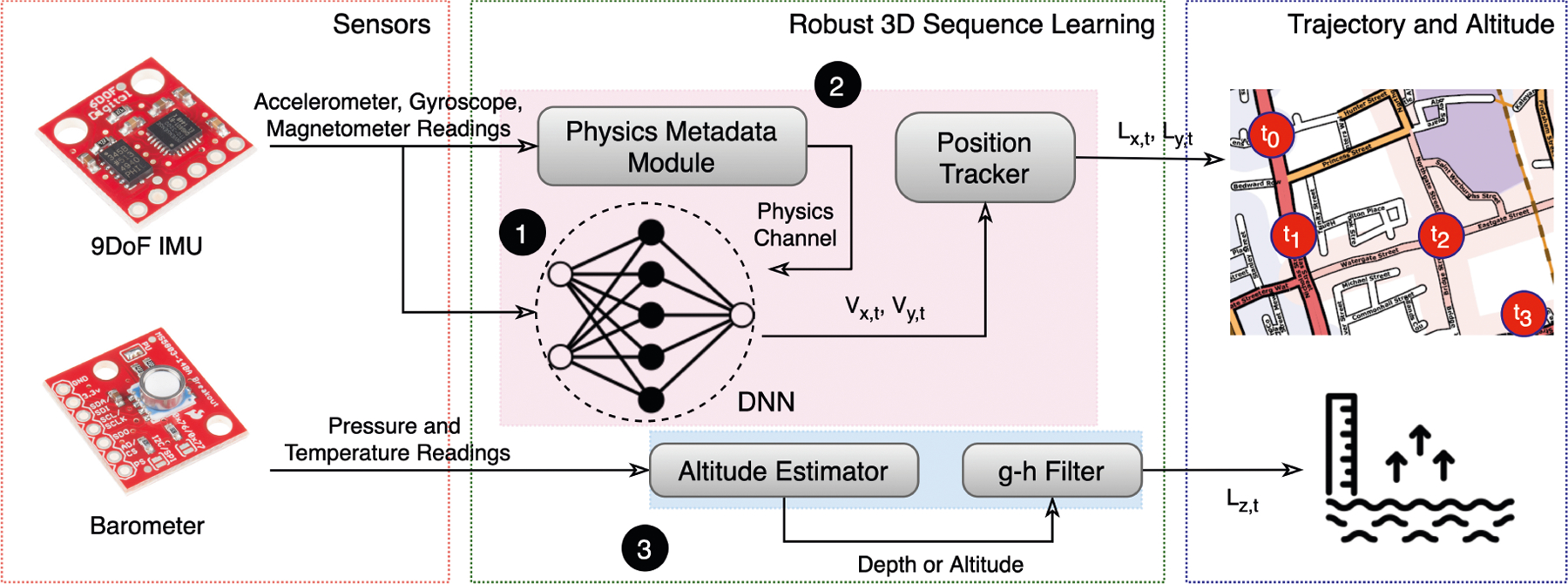
Components of robust 3D inertial sequence learning. (1) Velocity and magneto-centric DNN regresses velocities and uses magnetic North as an additional anchor point. (2) A physics metadata module that supplies latent information about whether valid translational movements have occurred or not from accelerometer readings. (3) A barometric g-h filter immune to inertial perturbations to regress altitude from pressure sensors.

To break the cycle of continuous integration and error propagation, IONet [16] proposed inertial sequence learning based on Newtonian physics. The goal is to estimate the change in navigation state over pseudo-independent inertial windows rather than absolute coordinates, constraining the ball of outputs for a neural network *f* to model. Under loose nonholonomic constraints and in polar coordinates, *f* is given as:

(9) $$\left(\text{Δ}l_{t},\text{Δ}\psi_{t}\right)=f_{\theta }\left(v^{I}(0),g_{0}^{I},{\hat{a}}_{\text{Δ}t}^{I},{\hat{w}}_{\text{Δ}t}^{I}\right)$$

where, Δ*t* = *t* : *t* – *n*, referring to a window of accelerometer ${\hat{a}}^{I}$ and gyroscope ${\hat{w}}^{I}$ samples of length *n*. The task involves estimating the initial velocity $v^{I}\left(0\right)$ and gravity $g_{0}^{I}$ in each window, which are treated as latent states. Instead, *f* outputs heading rate $\text{Δ}\psi_{t}$ and displacement $\text{Δ}l_{t}$ in the azimuthal plane. However, as we will showcase, the vanilla inertial sequence learning formulation suffers from gravity pollution, high-frequency inertial perturbations, varying sensor attitude, and heading rate singularity. We introduce a magnetometer, physics, and velocity-centric inertial sequence formulation robust to aforementioned problems, summarized in Fig. 2.

### 4.0.1 Latent Heading Information.

Gravity-aligned coordinate frames [46] are polluted by continuous translational motion due to the mixture of linear and gravitational acceleration. Gravity pollution can induce short-term offsets in the estimated orientation, leading to large velocity projection errors [70]. In addition, gyroscope BI and ARW generate long-term drift in the latent attitude estimate, further degenerating coordinate frame normalization in sensor fusion. As a result, we feed *f* with local magnetometer measurements ${\;}^{I}\hat{m}$ to provide additional latent information about device attitude and body heading, globally anchored by the 3D magnetic North **N**^*G*^. Magnetometers are motion-agnostic and do not suffer from long-term drift [70]. ${\;}^{I}\hat{m}$ provides an additional anchor $N_{0}^{I}$ to correct and constrain implicit estimation of $g_{0}^{I}$ and **v**^*I*^ (0) for each window, robust to varying sensor orientation, gravity pollution and continuous movements (e.g., circular trajectories). Furthermore, to emulate unrestricted sensor attitude and noise characteristics, we perform data augmentation during training via controlled random rotation **R** of inertial channels and addition of multivariate Gaussian noise 𝓝 [81]:

(10) $$s_{\text{Δ}t}^{x,y,z}\to Rs_{\text{Δ}t}^{x,y,z}+𝓝(0,\text{Σ})$$

### 4.0.2 Physics Metadata Channel.

Changes in sensor orientation and placement, hardware noise, ferromagnetic disturbances and body heading without linear movements can induce high-frequency inertial signatures, which can falsely trigger *f* to output invalid displacements. We supply *f* with latent valid motion metadata *c*_*t*_ (·) based on Newtonian kinematics to suppress the effects of high-frequency inertial artifacts. Specifically, we want *f* to be activated only when significant positional transitions have occurred. For humans, legged robots and terrestrial animals, *c*_*t*_ (·) corresponds to a local-variance step detector binary mask [71]:

(11) $$c_{t}\left({\;}^{I}\hat{a}\right)=\left\{\begin{array}{l} 1,{\hat{a}}_{L,\text{Δ}t}^{I}>\zeta \cdot \sqrt{\frac{\sum_{k\in \text{Δ}t}{\left({\hat{a}}_{L,k}^{I}-\bar{{\hat{a}}_{L,\text{Δ}t}^{I}}\right)}^{2}}{n}}\\ 0,\text{ otherwise } \end{array}\right.$$

where, ${\hat{a}}_{L,\text{Δ}t}^{I}=G_{5,f_{c}}\left(\left|{\hat{a}}_{\text{Δ}t}^{I}\right|\right)-G_{5,f_{c}}\left(|\bar{{\hat{a}}_{\text{Δ}t}^{I}|}\right)$, ζ is a tunable parameter and $G_{5,f_{c}}(\cdot )$ represents a 5th order low-pass filter with cutoff *f*_*c*_. For vehicles, *c*_*t*_ (·) signifies one of four transportation modes (stationary, accelerating, decelerating and constant speed) inferred through the discrete Fourier transform of $\left|{\;}^{I}{\hat{a}}_{\text{Δ}t}\right|$ [63]:

(12) $$c_{t}\left({\;}^{I}\hat{a}\right)=\underset{c_{t}(\cdot )}{\min}\left|\bar{\left|\text{FFT}\left(\left|{\hat{a}}_{\text{Δ}t}^{I}\right|\right)\right|}-\gamma_{k}\right|,k=\{1,2,3,4\}$$

where *γ*_*k*_ represents predefined threshold for *k*th transportation mode. The transportation mode metadata is important particularly to aid *f* better differentiate between constant velocity and stationary period inertial signatures.

### 4.0.3 Heading Rate Singularity.

The ground truth heading rate $\text{Δ}\psi_{t,g}$ is given as:

(13) $$\text{Δ}\psi_{t,g}=\psi_{t,g}-\psi_{t-n,g}$$

where,

(14) $$\left\{\psi_{t,g}=\text{mod}\left(\arctan2\left(\text{Δ}L_{y,g},\text{Δ}L_{x,g}\right),2\pi \right)\;\text{Δ}L_{i,g}=L_{i,g,t}-L_{i,g,t-n}\right.$$

*L*_*i,g*_ represents ground truth location. As $\text{Δ}L_{y,g}\wedge \text{Δ}L_{x,g}\to 0,\text{Δ}\psi_{t,g}↑$, leading to large spikes in heading rate. These outliers can severely degrade the performance of deep neural architectures [45] using mean squared error (MSE) loss. As a result, we modify the inertial sequence learning problem to regress *x* and *y* velocities rather than displacements and heading rates. Combined with latent heading and physics metadata channel, *f* is given as:

(15) $$\left(v_{x,t},v_{y,t}\right)=f_{\theta }\left(v^{I}(0),g_{0}^{I},N_{0}^{I},{\hat{a}}_{\text{Δ}t}^{I},{\hat{w}}_{\text{Δ}t}^{I},{\hat{m}}_{\text{Δ}t}^{I},c_{t}\left({\;}^{I}\hat{a}\right)\right)$$

We use strided velocity loss [46] for optimizing the parameters *θ* of *f* :

(16) $${𝓛}_{f}=E\left[{\left(v_{x,g,t}-v_{x,t}\right)}^{2}\right]+\kappa E\left[{\left(v_{y,g,t}-v_{y,t}\right)}^{2}\right]$$

The location at time *t* for sliding window with stride *s* and length *n* is given by:

(17) $$\left\{\begin{array}{c} L_{x,t}=L_{x,t-1}+\frac{s\cdot v_{x,t}}{n-s}\\ L_{y,t}=L_{y,t-1}+\frac{s\cdot v_{y,t}}{n-s} \end{array}\right.$$

### 4.0.4 Z-axis Dead-Reckoning.

Barometric sensors share many common coveted characteristics with inertial sensors [76], resulting in their widespread availability in current electronic systems [58]. We complement 2D sequence learning with altitude estimate by exploiting existing barometric chips to provide 3D dead-reckoning. We designed a model-free barometric *α* – *β* filter [8][86] with thermocline, salinity, noise and timestamp jitter mitigation [32]. The altitude measurements *L*_*z,m*_ at timestep *t* are given by:

(18) $$L_{z,t,m}=\left\{\begin{array}{l} {\left|-\frac{R\left(T_{c,t}+273.15\right)}{Mg}\ln\left(\frac{P_{t,\text{m}}}{P_{0}}\right)\right|}_{\text{air}}\\ {\left|\frac{P_{t,\text{m}}}{\rho_{0}g}\left(1-\frac{P_{t,\text{m}}}{K}\right)\right|}_{\text{fluid }} \end{array}\right.$$

where, *P*_*t,m*_ = pressure measurement, *M* = air molar mass, *1D454* = gravitational acceleration, *R* = gas constant, *T*_*c,t*_ = temperature in Celsius (from barometer) and *P*_0_= average sea level pressure (kPa). Furthermore:

(19) $$\rho_{0}=D\left(T_{c,t}\right)+sA\left(T_{c,t}\right)+s^{1.5}B\left(T_{c,t}\right)+cs^{2}$$

(20) $$K=E\left(T_{c,t},s\right)+F\left(T_{c,t},s\right)P_{t,\text{m}}+G\left(T_{c,t},s\right)P_{t,\text{m}}^{2}$$

where, $N\left(T_{c,t}\right)\leftarrow \gamma 10^{j}T_{c,t}^{k}$, $M\left(T_{c,t},s\right)\leftarrow \mu 10^{l}T_{c,t}^{n}s^{q}+N\left(T_{c,t}\right)$, *s* = salinity of fluid and *μ*, *γ*, *j*, *k*, *l*, *n* and *q* are constants. The filter prediction steps are given by:

(21) $$L_{z,t,p}=L_{z,t-1,p}+\text{Δ}T{\dot{L}}_{z,t-1,p},\;{\dot{L}}_{z,t,p}={\dot{L}}_{z,t-1,p}$$

The update steps are given by:

(22) $${\dot{L}}_{z,t,p}={\dot{L}}_{z,t,p}+\frac{\beta }{\text{Δ}T}\left(L_{z,t,m}-L_{z,t,p}\right)$$

(23) $$\underset{\text{altitude}}{\underset{︸}{L_{z,t,p}}}=L_{z,t,p}+\alpha \left(L_{z,t,m}-L_{z,t,p}\right)$$

${\dot{L}}_{z,t,p}$ refers to the vertical velocity of the object, *∆T* is the difference between current and previous timestamps and (*α*, *β*) are filter coefficients. Large values of *α* favor measurements over prediction, while large values of *β* increase the transient sensitivity of the filter. The barometric approach does not suffer from the effects of varying inertial sensor orientation and placement, gravity pollution, and unusual movements common in approaches using inertial sensors to regress height [59].

## 5 HARDWARE-AWARE INERTIAL NAVIGATION

In this section, we present the details of the neural network architecture adopted by TinyOdom (Section 5.0.1) and our NAS approach to enable deployments on URC devices (Section 5.0.2)

### 5.0.1 Backbone Neural Architecture.

We use a TCN [55, 78] to model *f* [10, 18, 35], which can jointly handle spatial and temporal features hierarchically. The receptive field *F*_*i*_ of each unit in the *i*th layer in a TCN dilated causal kernel of size *k* × *k* with dilation factor *l* is given by:

(24) $$F_{i,\text{TCN}}=F_{i-1}+\left(k_{l}-1\right)\times l,F_{0}=1$$

*F*_*i*_,_TCN_ is larger than *F*_*i*_,_TCN_, which is *i* × (*k* – 1) + *k.* Without explosion of parameter, memory footprint, layer count or overfitting, TCN kernels allow the network discover global context in long inertial sequences while maintaining input resolution and coverage. Causal convolutions maintain temporal ordering without requiring computationally intensive recurrent units, supporting out-of-order parallelization during training. In addition, two stacks of dilated causal convolution layers are fused through gated residual blocks **z** for expressive yet bounded non-linearity, complex interactions and temporal correlation modeling in the input sequence:

(25) $$z=\tanh\left(W_{f,k}*x\right)⊙\sigma \left(W_{g,k}*x\right)$$

### 5.0.2 Neural Architecture Search.

To find the ideal neural inertial candidate from the backbone TCN for limited flash, RAM, and latency requirements, we model the search as a parallelizable black-box BO problem. The search space Ω consists of neural network weights *w*, hyperparameters *θ*, network structure denoted as a directed acyclic graph (DAG) *g* with edges *E* and vertices *V* representing activation maps and common ML operations *ν* (e.g., convolution, batch normalization, pooling, etc.) respectively, which act on *V*. The goal is to find a neural network that maximizes the hardware SRAM and flash usage within the device capabilities while minimizing latency and validation RMSE.

(26) $$f_{\text{opt }}=\lambda_{1}f_{\text{error }}(\text{Ω})+\lambda_{2}f_{\text{flash }}(\text{Ω})+\lambda_{3}f_{\text{SRAM }}(\text{Ω})+\lambda_{4}f_{\text{latency }}(\text{Ω})$$

where

(27) $$f_{\text{error }}(\text{Ω})={𝓛}_{\text{validation }}(\text{Ω}),\text{Ω}=\{\{V,E\},w,\theta ,v\}$$

(28) $$f_{\text{flash }}(\text{Ω})=\left\{\begin{array}{l} -\frac{{\left\|h_{\text{FB}}(w,\{V,E\})\right\|}_{0}}{{\text{ flash }}_{\max}}\vee -\frac{\text{ HIL information }}{{\text{ flash }}_{\max}}\\ \infty ,f_{\text{flash }}(\text{Ω})>{\text{ flash }}_{\max} \end{array}\right.$$

(29) $$f_{\text{latency }}(\text{Ω})=\frac{\text{ FLOPS }}{{\text{ FLOPS }}_{\text{target FLOPS }}}\vee \frac{\text{ HIL information }}{{\text{ Latency }}_{\text{target latency }}}$$

(30) $$f_{\text{SRAM}}(\text{Ω})=\left\{\begin{array}{l} -\frac{\max_{l\in [1,L]}\left\{{\left\|x_{l}\right\|}_{0}+{\left\|a_{l}\right\|}_{0}\right\}}{{\text{SRAM}}_{\max}}\vee -\frac{\text{ HIL information }}{{\text{SRAM}}_{\max}}\\ \infty ,f_{\text{SRAM }}(\text{Ω})>{\text{SRAM}}_{\max} \end{array}\right.$$

$$a=w\vee y,\;y=\sum_{k=1}^{K}v_{k}g_{k}\left(x,w_{k}\right)$$

The objective function *f*_opt_ can be thought of as seeking a Pareto-optimal configuration of parameters Ω^∗^ under competing objectives [30] such that:

(31) $$f_{k}\left({\text{Ω}}^{*}\right)<=f_{k}(\text{Ω})\;\forall k,\text{Ω}\;\wedge \exists j:f_{j}\left({\text{Ω}}^{*}\right)<f_{j}(\text{Ω})\;\forall \text{Ω}\neq {\text{Ω}}^{*}$$

We use Gaussian process as the surrogate model to approximate *f*_opt_, which allows priors on the distribution of moments to propagate forward as the search progresses. In addition, the domain of random scalarizations *λ* can be specified by the user to guide the parallel search acquisition functions (hallucination or K-means clustering) into the promising Pareto-optimal regions of the gradient plane. The acquisition function decides the next set of Ω_*n*_ to sample from the design space using Monte Carlo sampling with Bayesian Upper-Confidence Bounds (UCB), also known Thompson sampling, which balances exploration and exploitation [68]. Apart from speeding up the NAS, parallel search ensures that NAS is not being performed on network morphs early on (exploitation) and information gain is maximized in the search process (exploration), yielding a stage-wise “coarse-to-fine” search space:

(32) $$\hat{f}(\text{Ω})∼𝓖𝓟\left(\mu (\text{Ω}),k\left(\text{Ω},{\text{Ω}}^{\prime }\right)\right)$$

(33) $${\text{Ω}}_{t}=\arg\underset{\text{Ω}}{\max}\left(\mu_{t-1}(\text{Ω})+\beta^{0.5}\sigma_{t-1}(\text{Ω})\right)$$

**Fig. 3.**
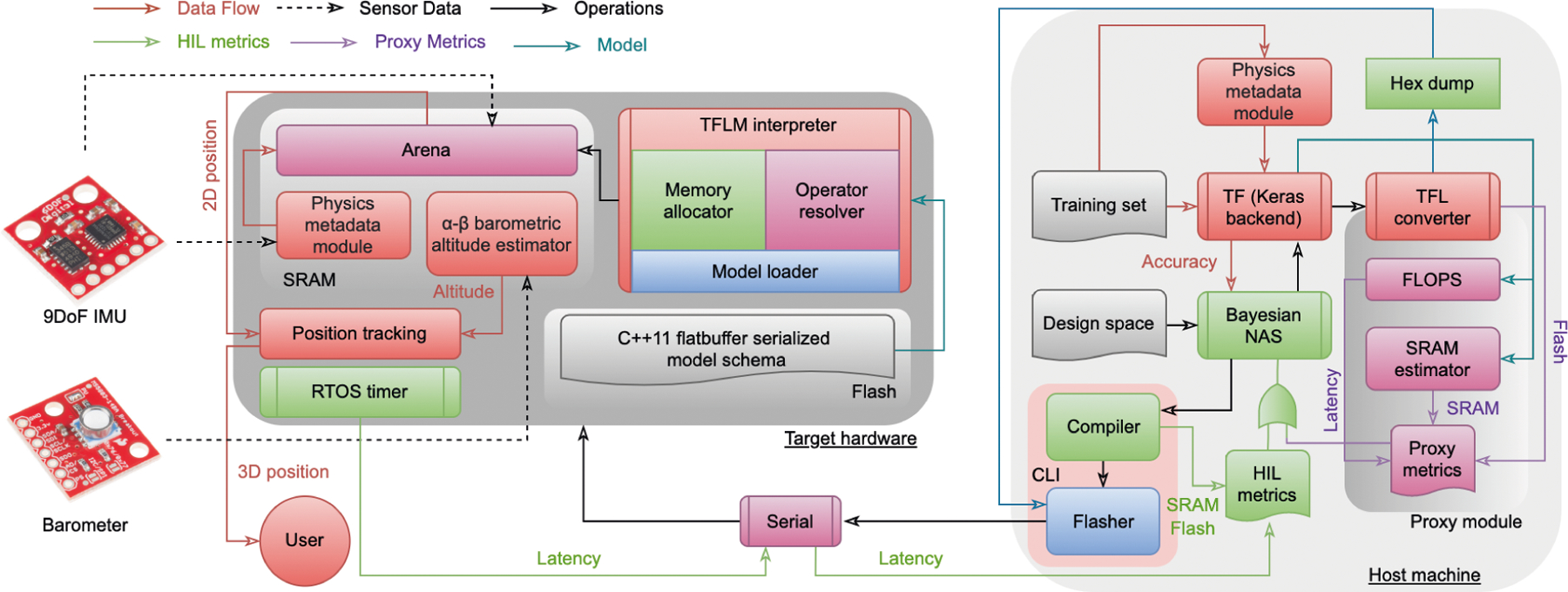
Implementation of hardware-aware neural-inertial navigation. The framework supports both the use of proxy and real-hardware to get hardware constraint estimates.

Firstly, validation RMSE serves as a proxy for the error characteristics *f*_error_ (Ω) of the model candidate. Secondly, when real hardware is absent, we use the size of the flatbuffer model schema *h*_FB_ (·) [23] as a proxy for flash usage. Thirdly, we use the standard RAM usage model as a proxy for SRAM usage *f*_SRAM_ (Ω), with intermediate layer-wise activation maps and tensors being stored in SRAM [30]. Lastly, since model latency is linearly proportional to the FLOPS count for a variety of convolutional models for microcontrollers, we use FLOPS as a proxy for runtime latency *f*_latency_ (Ω) [6]. When HIL is available, we obtain the SRAM, flash, and latency parameters directly from the target compiler and real-time operating system (RTOS). All hardware parameters are normalized by device capacity or target metrics. The entire NAS pipeline is summarized in Fig. 3.

## 6 EXPERIMENTAL SETUP

In this section, we provide details on the implementation of our hardware-aware robust 3D sequence learning framework. We list the domain space of the TCN backbone (Section 6.1) to be optimized by NAS. Next, we provide details on how we setup BO in Python (Section 6.2). Then, we outline the datasets (Section 6.3) used to train and benchmark the performance TinyOdom models and competing baselines (Section 6.4). Afterward, we list the performance metrics used for benchmarking (Section 6.5). Next, we provide details on the target hardware, host machine, and software specifications (Section 6.6). Finally, we provide details on our real-world experimental setup (Section 6.7).

### 6.1 NAS Search Space

Our TCN model consists of an input layer, followed by the TCN backbone. The hyperparameters to be optimized by the NAS framework for the TCN backbone are as follows:

- Number of filters: 2–64
- Kernel size: 2–16
- Use of residual (skip connections): True, False
- Number of layers: 3–8
- Dilation factor to assign to each layer: [1, 2, 4, 8, 16, 32, 64, 128, 256]
- Dropout: 0.0–1.0
- Normalization: Weight, Layer, Batch

The fixed parameters for the TCN backbone are as follows:

- Number of stacks: 1
- Activation: ReLU
- Learning Rate: 0.001 (Adam)

The outputs of the TCN backbone are reshaped, pooled, and flattened. The flattened vectors are fed to a 32 unit fully-connected layer. The final output of the TCN model is x and y velocities.

### 6.2 NAS Implementation

Our NAS implementation is based on the state-of-the-art open-source BO library called Mango [68][69]. Our NAS implementation consists of three steps: (i) NAS search space definition, (ii) multi-objective function specifications, and (iii) hardware-in-the-loop or proxy constraints computation. Our NAS search space is a combination of categorical, integer, and continuous variables as shown in Section 6.1. This search space is realized using python constructs (lists, dictionaries) and SciPy [79] distributions which are directly supported in Mango [69]. We create *f*_opt_ (Equation 26) in Python, where the hardware metrics are computed either using proxies or hardware-in-the-loop. For training TCN models for each application, we run the BO search strategy for 50 iterations. The internal surrogate model used by our implementation is based on the Gaussian process [69] which uses the upper confidence bound as the acquisition function. The surrogate model approximates the hyperparameter decision boundary learning the best regions that minimize the *f*_opt_. The next sampled hyperparameter is selected based on the predicted mean (exploitation) and the corresponding variance (exploration) which are included as part of the acquisition function. In our implementation, we use the adaptive exploitation versus exploitation trade-off and automatic domain size explorations from Mango [68][69].

### 6.3 Benchmark Datasets

To train the TCN models and evaluate the performance of TinyOdom against competing proposals, we selected five inertial odometry datasets that have been widely used to benchmark existing inertial dead-reckoning techniques for various applications [16, 18, 29, 31, 46, 88]. Table 1 summarizes the representative characteristics of the five datasets. For PDR, we selected the OxIOD [18] and the RoNIN [46] dataset, the two largest publicly available inertial odometry datasets for human localization. Both OxIOD and RoNIN use smartphone inertial sensors to collect 9DoF IMU data. However, the RoNIN dataset assumes unrestricted phone orientation and phone placement to support natural day-to-day smartphone usage and provides a more challenging task of developing inertial models invariant to device placement or orientation. Furthermore, the trajectories in the RoNIN dataset have a larger spatial span compared to the trajectories in the OxIOD dataset. The OxIOD dataset, on the other hand, has a higher ground truth resolution (sub-mm) thanks to the use of a Vicon motion capture setup.

For UUV, UAV, and animal localization, we chose the AQUALOC [31], the EuRoC MAV [13], and the GunDog [41] dataset, the only publicly available datasets for benchmarking dead-reckoning algorithms for UAV, UUV, and animals. The EuRoC MAV, AQUALOC, and the GunDog datasets collect IMU data from sensor tags fixed to quadrotors, underwater probes, and penguins, respectively. The size of these datasets is much smaller than the OxIOD and RoNIN datasets. We used the AQUALOC dataset to evaluate the performance of the barometric altitude estimator besides 2D inertial tracking, as it includes underwater pressure sensor data. The only caveats in these three datasets are the lack of magnetometer and gyroscope readings in the EuRoC MAV and the GunDog dataset, respectively. We adapted our TCN input layers to work with the available sensors in these two datasets.

Table 2 lists the window size, stride, data splits, and epochs that we used in the training pipeline for our TCN model for each dataset. We split the datasets by sequences (separate files). We did not use any validation during the training phase of each candidate model but rather used the validation split to compute the error metric *f*_error_ (Ω) in the outer loop of the NAS. The test split was used in the final evaluation of the best-performing model found via NAS against baseline techniques. For the OxIOD and RoNIN dataset, we used the same window size and stride used by IONet [16], L-IONet [18], and RoNIN [46] on the two datasets. For the EuRoC MAV and GunDog dataset, we chose a window size of 0.25 seconds to account for the faster maneuverability of drones and penguins over humans. On the contrary, since underwater vehicles move slowly, we used a window size of 2 seconds for the AQUALOC dataset. Note that the dataset splits are different from each other because the datasets were split by sequences/files and not samples to preserve continuous trajectories. For the RoNIN dataset, we used splits provided by the dataset makers [46]. For the OxIOD, the AQUALOC, and the EuRoC MAV datasets, we split the files such that the training set, the validation set, and the test set roughly have 80%, 5%, and 15% of the total dataset samples respectively. Since there are only two complete trajectories in the GunDog dataset, we split the training trajectory into a training and validation split, while using the other trajectory as a test split.

### 6.4 Baseline Algorithms

To evaluate the utility of TinyOdom, we use several SOTA inertial odometry techniques as baselines for the four applications. For tracking humans via the OxIOD and the RoNIN datasets, we use the following baselines:

#### Step Detector with Weinberg SLE (PDR):

We used one of the PDR algorithms proposed by Jimenez *et al.* [48]. The algorithm uses a threshold-based step detector based on accelerometer peaks and updates the displacement via Weinberg Stride Length Estimation (SLE) [85], which models step length in terms of vertical movement of the pelvis during each step. The heading is computed from the gyroscope. PDR is one of the most widely used classical inertial localization method [44, 91]. We used a publicly available implementation the PDR ^2^

#### Naive Double Integration (NDI):

We use the ideal formula for dead-reckoning (Equations (5–7)), where we simply integrate the linear accelerometer readings to get the position after coordinate transformation [88]. We used a publicly available implementation of the NDI^2^.

#### IONet:

IONet [16] is the first deep inertial sequence learning model. The LSTM model uses the original heading-displacement formulation of neural inertial localization and takes in gyroscope and accelerometer readings as input. IONet was shown to outperform PDR and strap-down inertial navigation system (SINS) on the OxIOD dataset. We implemented our own version of IONet using the same architectural encodings mentioned in [16], as the code is not publicly available.

#### L-IONet:

L-IONet [18] improves the computational efficiency over IONet by using a TCN in place of LSTM, without significantly sacrificing localization performance. We implemented our own version of L-IONet, as the code is not publicly available.

#### RoNIN TCN:

RoNIN TCN [46] uses robust velocity loss and a heading-agnostic coordinate frame to account for unrestricted sensor orientation and placement. RoNIN TCN outperformed NDI, PDR, and IONet on the RoNIN and OxIOD datasets. We used the publicly available implementation of RoNIN TCN ^3^ for retraining and benchmarking.

For UUV localization using the AQUALOC dataset, we use the following baseline:

#### NavNet:

NavNet [96] asynchronously combines inertial sensor and doppler velocity log (DVL) through separate LSTM networks, followed by attention layers to capture relevant long-term contextual information across timesteps and regress x and y velocities. NavNet was shown to outperform EKF and UKF for underwater localization. We implemented our own version of NavNet, as the code is not publicly available.

For UAV localization using the EuRoC MAV dataset, we use two baselines:

#### AbolDeepIO:

AbolDeepIO [29] uses two separate LSTM channels to map accelerometer and gyroscope readings to different latent representations while feeding the sampling rate of the inertial sensor through a third LSTM channel to make the displacement and heading regressor robust to sampling rate jitter. The high-level correlated features are then fused using slow fusion. AbolDeepIO was shown to outperform VINet [19], SINS, and IONet. We implemented our own version of AbolDeepIO, as the code is not publicly available.

#### VeTorch:

VeTorch [35] uses two TCN to compute the heading and displacement of autonomous vehicles from smartphone accelerometer and gyroscope readings. It also transforms inertial dynamics from the phone to the car. VeTorch was shown to outperform EKF and IONet. We implemented our own version of VeTorch, as the code is not publicly available.

Finally, for animal tracking, we use the following baseline:

#### GunDog:

GunDog [41] complements the ideal formula for dead-reckoning (Equations (5–7)) with a step detector and compass calibration to constrain error explosion during animal tracking. We used the publicly available implementation of GunDog ^4^.

### 6.5 Performance Metrics

We adopt two widely used [46, 59, 97] metrics to quantify the localization performance:

- **Absolute Trajectory Error (ATE)**: ATE is defined as the average root-mean-squared-error (RMSE) between the actual and the predicted locations for the entire trajectory [46]:
(34) $$ATE=\frac{1}{T}\sum_{t\in T}\sqrt{{\left(L_{x,t}-L_{x,g,t}\right)}^{2}+{\left(L_{y,t}-L_{y,g,t}\right)}^{2}}$$
  The lower the ATE, the better.
- **Relative Trajectory Error (RTE)**: RTE is defined as the average root-mean-squared-error (RMSE) between the actual and the predicted locations for a specific time interval. Inspired from [46], we use a time interval of 1 minute to calculate RTE. The lower the RTE, the better.

To quantify the resource usage of the proposed dead-reckoning techniques, we use either the size (flash usage) of the TFLM flatbuffer serialized model schema for neural-inertial models or use the flash usage of the compiled embedded C code for classical techniques.

### 6.6 Hardware and Software Specifications

For benchmarking HIL NAS, we use three real ARM Cortex-M target boards and one virtual hardware model (proxy) with varying resource constraints. The target hardware specifications are summarized in Table 3. The processor runs Mbed RTOS and TFLM interpreter on-board. To communicate with the target hardware via system commands from the host machine, we used the Mbed command-line interface (CLI).

The TinyOdom models were implemented in Jupyter Notebook (Python), using Keras via a Tensorflow and TFLM [23] backend. All the publicly-unavailable baselines were also implemented similarly. To benchmark PDR, NDI, and GunDog’s resource usage, we rewrote their MATLAB and R code in C++. Table 4 lists the specifications of the host machines on which we ran the NAS and model training. Our NAS framework supports training on machines with a wide range of processor, GPU, and RAM configurations.

### 6.7 Real-World Setup

**Fig. 4.**
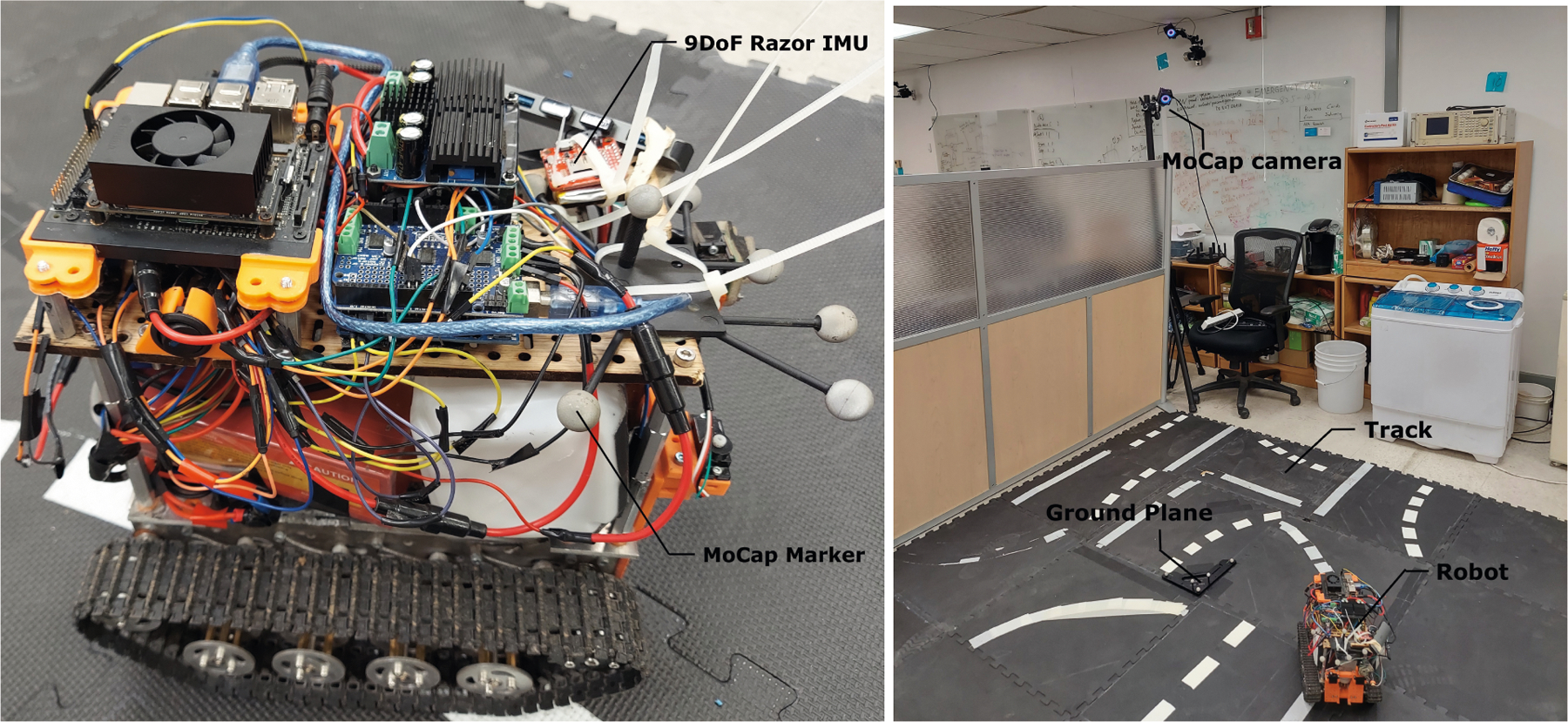
Setup for testing the utility of TinyOdom in the real-world. A 9DoF Razor IMU board logs inertial sensor data from an agricultural robot onto an SD card, with developed neural-inertial model running in real-time on the board after training. Motion capture (MoCap) markers are attached to the robot to log ground truth position on the track using high-resolution infrared cameras with respect to the ground plane.

To showcase how TinyOdom can support real-world applications, we retrofitted an agricultural robot intended for precision farming [27][28] with neural-inertial tracking hardware and software. The setup is shown in Fig. 4. The robot is intended for autonomous inter-row weed control in flaxseed and canola fields, where the spacing between adjacent crop lines can be as small as 30 cm. Thereby, the robot requires a high-sampling rate cm level precision localization [27][28], which is not possible to achieve with commodity GPS alone. We attached a 9DoF Razor IMU board to the robot to perform inertial data logging as well as on-board neural-inertial tracking. The board features a MEMS accelerometer (ADXL345), a gyroscope (ITG-3200), and a magnetometer (HMC5883L). The data is logged onto an SD card. The board features 32kB of SRAM and 256 KB flash. To log sub-mm ground truth position, we used several OptiTrack Prime 17W^5^ MoCap infrared markers [34] mounted in a rigid body configuration. The motion data of the rigid body were tracked using Motive:Tracker^6^ [4]. To synchronize the ground truth data and the IMU data, we harmonized the local system clocks to the Network Time Protocol and also performed graphically identifiable special movements with the robot before collecting position data. We had two experimental phases with the robot:

- **Data Collection Phase**: In this phase, we drove the robot within a 2×2 m arena using a remote controller to collect inertial sensor data and ground truth position data. We collected 3 hours of IMU and ground truth position data at a 100 Hz sampling rate.
- **Evaluation Phase**: In this phase, we ported a neural-inertial model on the Razor IMU platform. Instead of logging IMU data this time, the board logged the estimated position of the robot. The driving patterns and ground truth collection setup remained the same.

## 7 EVALUATION, COMPARISON AND DISCUSSION

In this section, first, we showcase the localization performance and resource usage of TinyOdom models against competing baselines (Section 7.1). Next, we illustrate how our HIL NAS adapts models based on hardware capability (Section 7.2). Third, we show the performance of our depth filter for altitude estimation (Section 7.3). Fourth, we perform an ablation study to show how the individual components in our physics, velocity, and magnetometer-centric sequence learning formulation affect localization performance (Section 7.4). Finally, we show transferability and real-world evaluation of TinyOdom (Section 7.5).

### 7.1 Localization Performance and Resource Usage

**Fig. 5.**
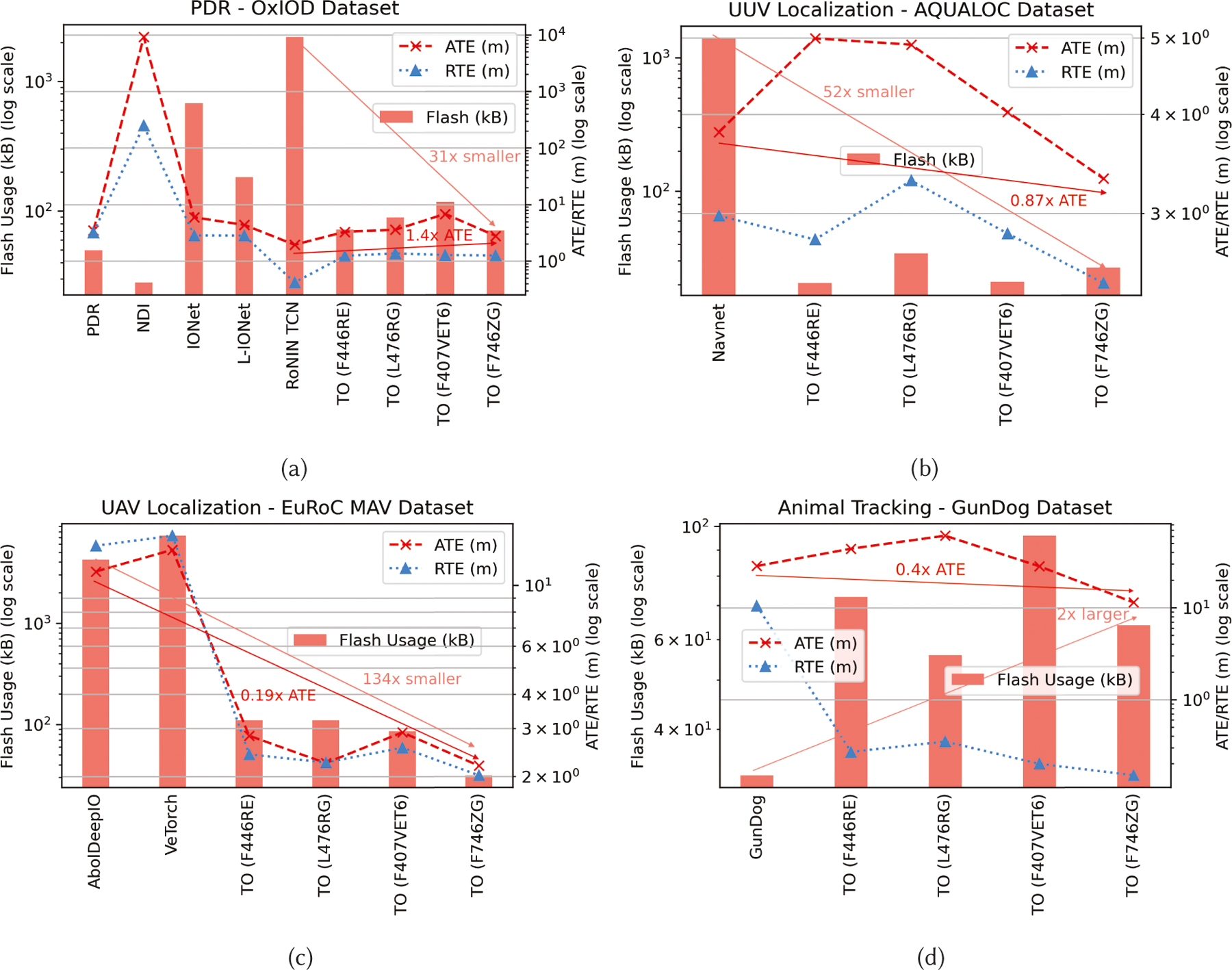
ATE, RTE, and flash usage (lower is better) of competing dead-reckoning techniques against TinyOdom (TO) models. TinyOdom can provide 31–134 reduction in model size without significant markup (often improvement) in ATE/RTE over the SOTA methods in each application, thanks to robust sequence learning and hardware-aware formulation.

Table 5 and Fig. 5 showcase the performance of TinyOdom TCN models against competing methods for all four applications in terms of ATE, RTE, and resource usage. From Table 5 and Fig. 5, we can see that TinyOdom models, despite being a fraction of the size of competing baselines, are always among the top two best performing models in terms of ATE and RTE. Specifically, TinyOdom outperforms the SOTA for UUV, UAV, and animal tracking by reducing the ATE by 1.14× to 5×, while being 52× to 134× lighter. Classical approaches such as PDR, NDI, and Gundog are outperformed not only by TinyOdom but other deep-learning neural inertial models. Notice how our best performing models are within 27–72 kB, while the best performing baselines are 33–2200 kB. For human localization, TinyOdom competes with RoNIN TCN while outperforming NDI, PDR, IONet, and L-IONet. Compared to the RoNIN TCN, the top-performing TinyOdom models are 31× to 34× smaller, while overall, the framework provides 31× to 134× reduction in model size over the SOTA. We can make several important inferences from Table 5 and Fig. 5:

- Baselines which regress heading and displacement (e.g., IONet, L-IONet, AbolDeepIO, and VeTorch) have high ATE and RTE. We observed that the errors build up when sharp turns occur in the trajectory. Given that objects in these datasets mostly travel in straight lines, the singularities in the heading rates are ignored by the neural network during training, leading to large errors in the final position estimate.
- Note that the ATE and RTE for all techniques are much higher than RoNIN TCN for the RoNIN dataset. We hypothesize that this happened because the pre-trained RoNIN TCN was trained on the entire RoNIN dataset, while we only had access to 50% of the data (which is challenging due to unrestricted sensor configuration). In fact, the performance of RoNIN TCN is similar to TinyOdom on the OxIOD dataset, with TinyOdom lagging RoNIN TCN by 0.85 m. The performance gain comes from the 34× more weights available to the RoNIN TCN. RoNIN TCN also relies on device orientation, which itself is polluted.
- The robust sequence learning and hardware-aware formulation of TinyOdom generalize across heterogeneous applications, continuously maintaining superior localization resolution while keeping a low resource overhead. TinyOdom attempts to lower the ATE and RTE based on available resources. Observe from Fig. 5 that with more available memory, the ATE and RTE generally go down.
- Excluding PDR, NDI and GunDog and L-IONet, none of the baselines are suitable for deployment on URC devices due to memory constraints. All 4 of these baselines are outperformed in localization resolution.

**Fig. 6.**
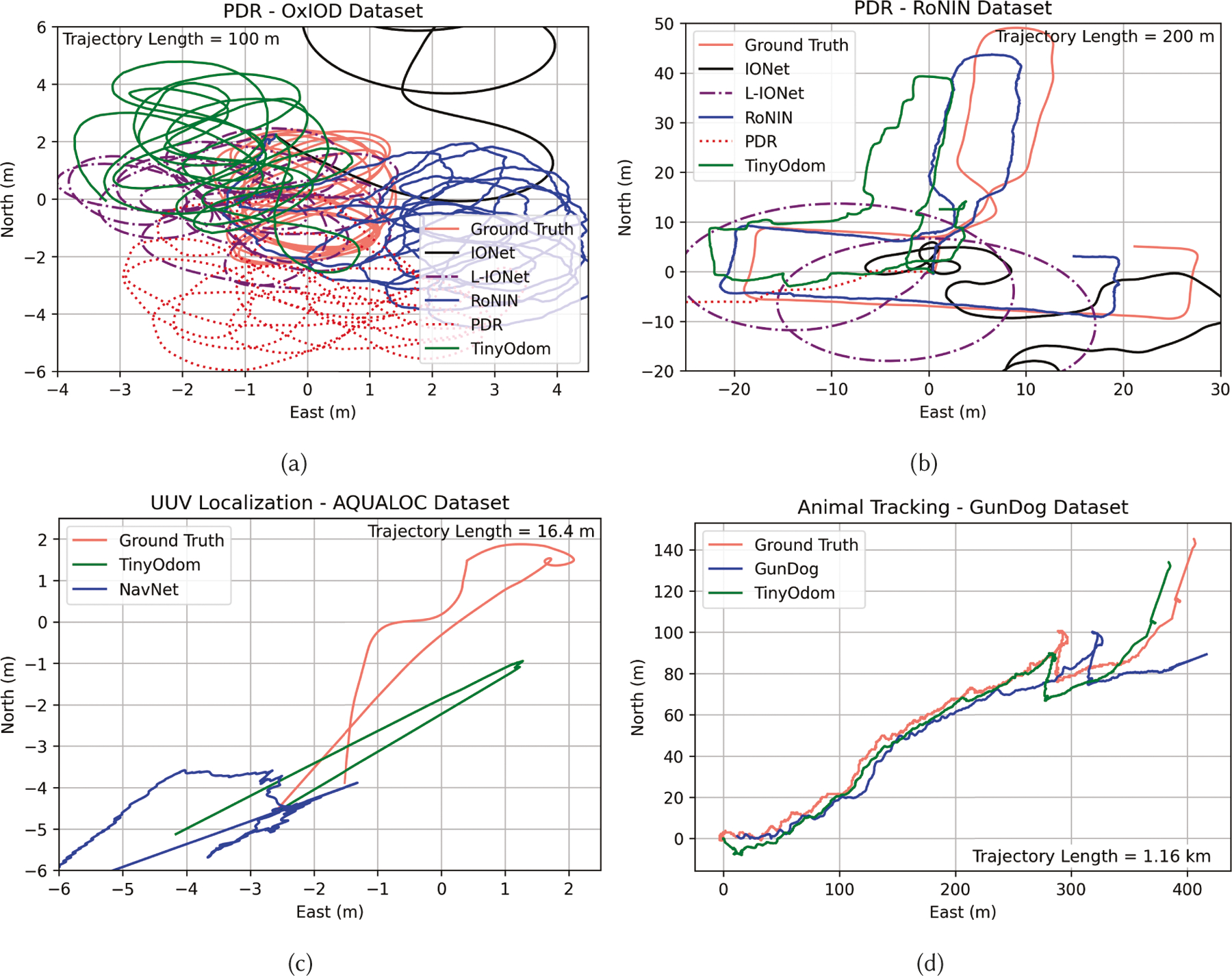
Selected trajectory reconstructions on unseen test data from (a) OxIOD dataset, (b) RoNIN dataset, (c) AQUALOC dataset, and (d) GunDog dataset, in the NE coordinate frame, for TinyOdom and competing proposals.

Fig. 6 shows selected trajectory reconstructions of varying lengths by TinyOdom models against competing methods for PDR, UUV, and animal tracking. From Fig. 6, we can see that TinyOdom can perform dead-reckoning for varying trajectory lengths without explosion of position estimation error. Due to heading rate singularity, IONet and L-IONet struggle to constrain the errors generated by sharp turns, evident in Fig. 6(b), where IONet and L-IONet tend to over-smooth the turns in the trajectory. PDR has a large error radius and completely fails on the RoNIN dataset, where the heading cannot be inferred via model-based techniques because of unrestricted phone placements. TinyOdom models are more closely able to replicate the trajectories generated by RoNIN TCN with 31×–34× lower model size. For UUV and animal tracking, TinyOdom generates trajectories that closely mimic the ground truth, while baselines either fail or are further apart from the ground truth.

**Fig. 7.**
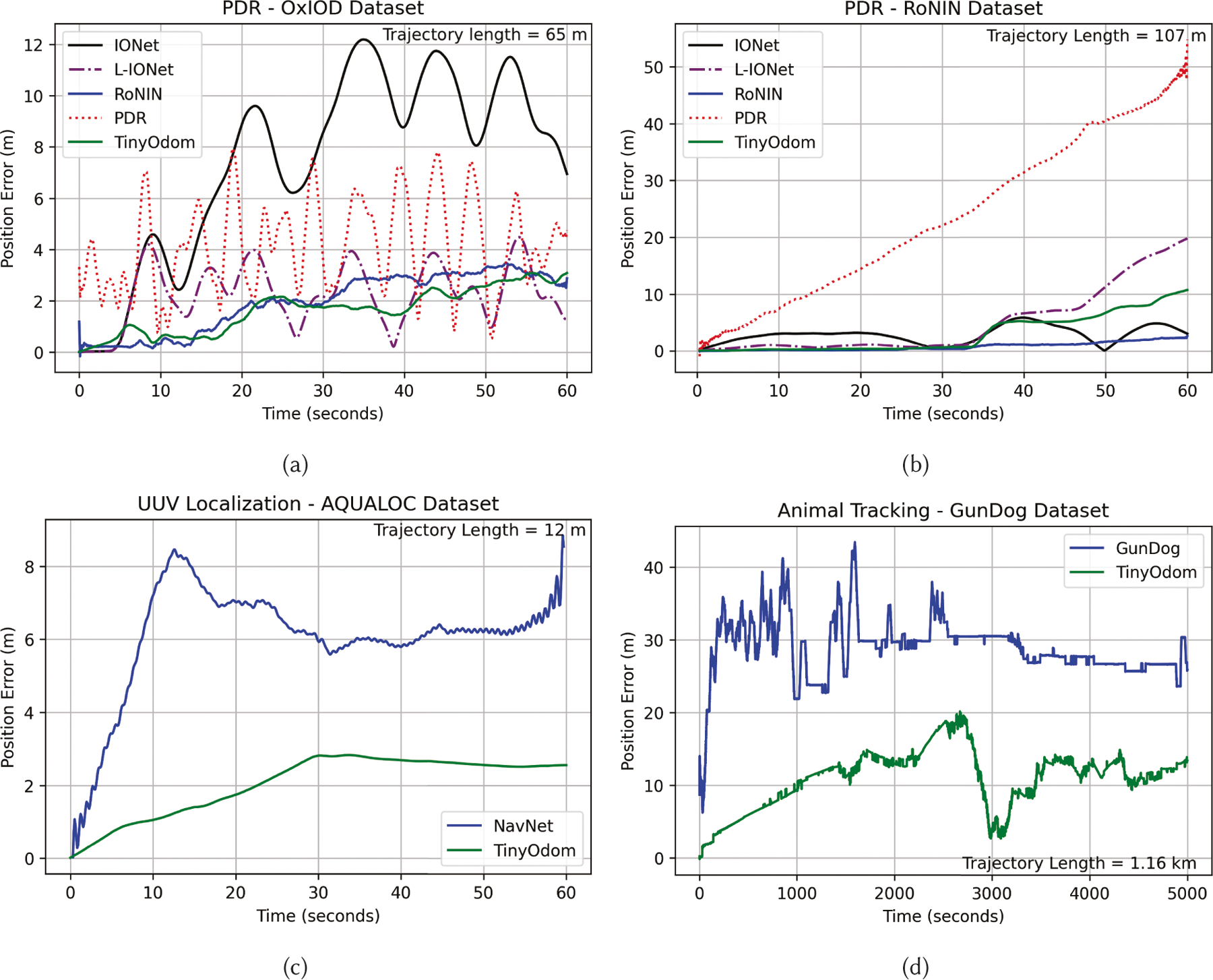
Evolution of position estimation error with time on selected unseen trajectories from (a) OxIOD dataset, (b) RoNIN dataset, (c) AQUALOC dataset, and (d) GunDog dataset, for TinyOdom and competing proposals.

For small trajectories, the average ATE and RTE metric does not completely state how the error evolves with time [97]. For example, if a trajectory is circular with a radius of a few meters (common in the OxIOD dataset [18]), the average ATE and RTE would be unable to showcase how the location estimation varied with time. As a result, we evaluated how the position estimation error evolves with time for PDR, UUV, and animal tracking for all methods, shown in Fig. 7. From Fig. 7, we can observe that the position estimation error of TinyOdom grows much more slowly with time compared to baseline techniques. TinyOdom provides position estimate within 2.5m to 12m for trajectories of length 12m to 1160m spanning 60 seconds. Notice how the error of the PDR, IONet, and L-IONet is sinusoidal with time in the OxIOD dataset while growing linearly in the case of the RoNIN dataset. Since movement is constricted in a limited space for circular trajectories, ATE of these three baselines fluctuates for the OxIOD dataset even as they provide poor position estimation. On the other hand, the error of RoNIN-TCN, NavNet, GunDog, and TinyOdom grow linearly with time.

### 7.2 Evaluation of Hardware-in-the-Loop Bayesian Neural Architecture Search

**Fig. 8.**
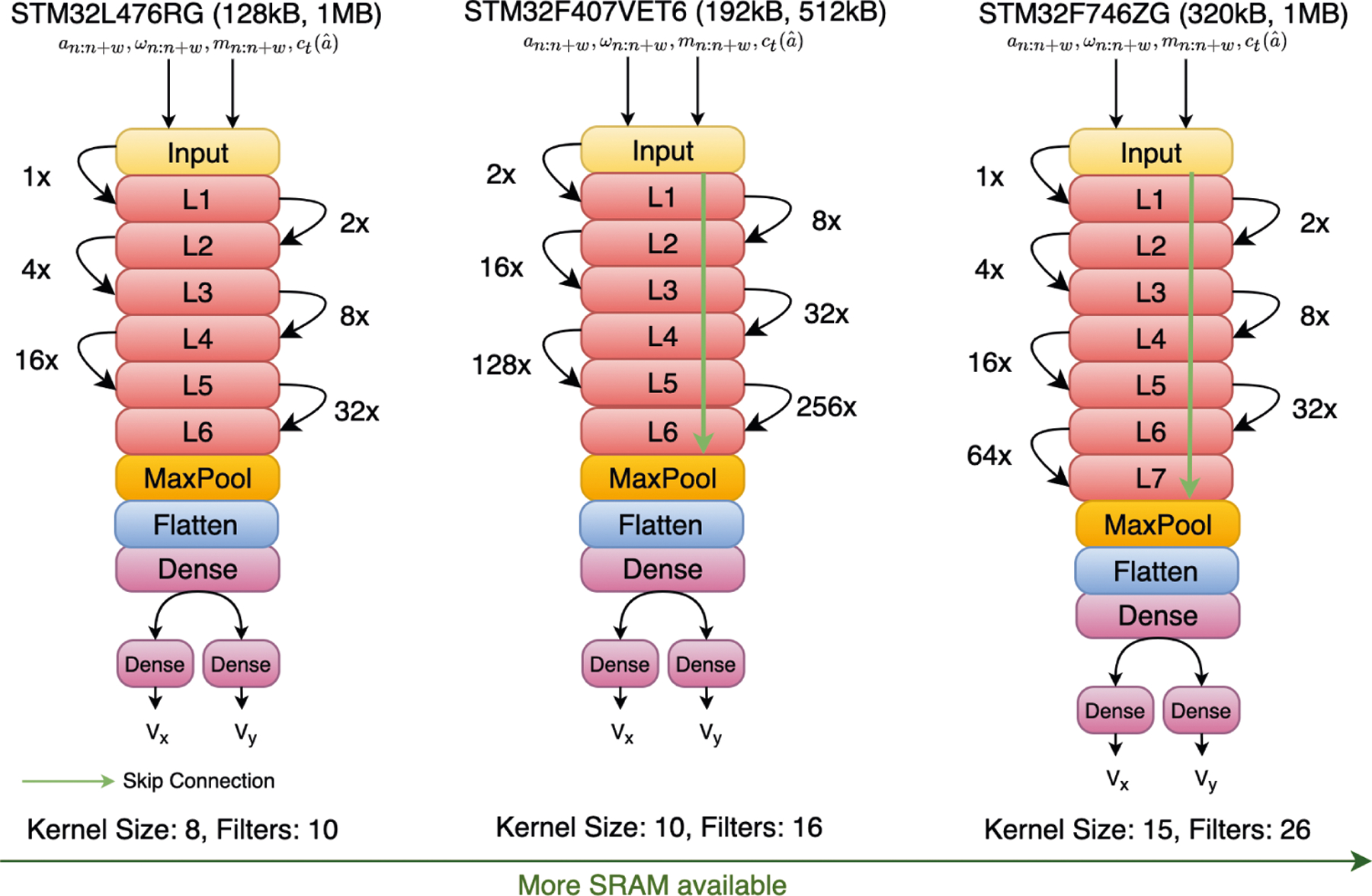
Architectural adaption and device capability exploitation by Bayesian NAS based on resource usage for TinyOdom models. The RAM and flash constraints of the device are written inside paranthesis. *L*_*i*_ refers to *i*^th^ layer of TCN.

Fig. 8 showcases how our hardware-aware NAS adapts architectural encodings of the TCN backbone for hardware with different compute constraints for the RoNIN dataset. Instead of simply providing small models every time, our NAS framework optimizes the TCN model to maximize resource usage, thereby lowering ATE and RTE, when more resources are available. For example, in Fig. 8, as the SRAM capacity increases, the NAS framework also increases the number of layers, filters, and the kernel size of the TCN models. The framework even adds skip connections to prevent exploding and vanishing gradient problems for deeper networks. In addition, to capture both local and long-term inter-dependencies in the temporal sequence within a limited computing budget, our NAS framework assigns small dilation factors to lower layers and large dilation factors to higher layers. This is counter-intuitive, as human designers would normally assign dilation factors that grow by a constant factor with each successive layer instead of intelligent assignment performed by NAS.

**Fig. 9.**
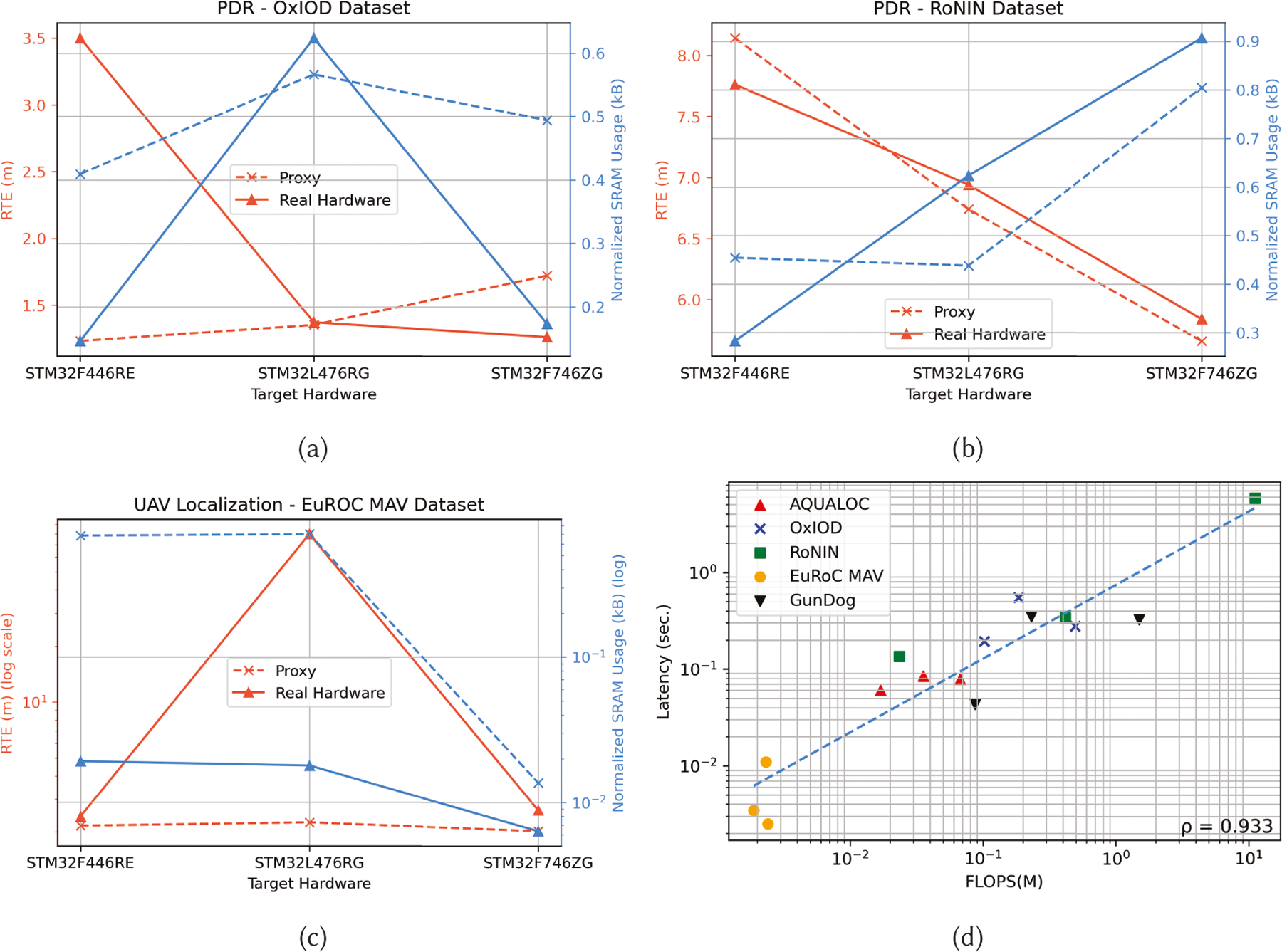
(a)-(c) RTE and SRAM usage estimation comparison between proxyless Bayesian NAS and proxied Bayesian NAS for different devices. The SRAM usage is normalized by maximum RAM capacity of each device. (d) Relationship between FLOPS and model latency for models trained on all five datasets with HIL.

We also performed an ablation study to see how proxyless (with real-hardware) and proxied versions (with proxy to simulate hardware metric) of our NAS framework differ in performance with three real hardware devices for PDR and UAV localization. The results are showcased in Fig. 9 (a)(b)(c). From Fig. 9 (a)(b)(c), we can see that the proxy generally tends to provide models which need a higher SRAM compared to HIL NAS. This is because the HIL NAS can take into account the model runtime interpreter and RTOS overhead, which the proxy for SRAM and flash fails to account for. Thus, some well-performing models (models with low ATE) found by proxied NAS may not fit on the real hardware. Thus, HIL is especially important for URC devices, where all overheads need to be accounted for. Besides quantifying the difference between proxyless and proxied NAS for memory and RTE modeling, we also studied the relationship between FLOPS and model latency (from real hardware) for all five datasets, with the results summarized in Fig. 9(d). From Fig. 9(d), we can observe that even though the models were trained on widely different datasets, there is still a strong positive correlation (Pearson Coefficient, *ρ* = 0.933) between FLOPS and model latency in the log-log scale, indicating that it is possible to develop an analytical model correlating FLOPS and model latency without requiring HIL for explicit latency modeling. This would save time as the models do not need to be run on real hardware to benchmark latency but would only require compilation to get the SRAM and flash, which can be achieved on the host machine.

### 7.3 Performance of Robust Depth Filter

**Fig. 10.**
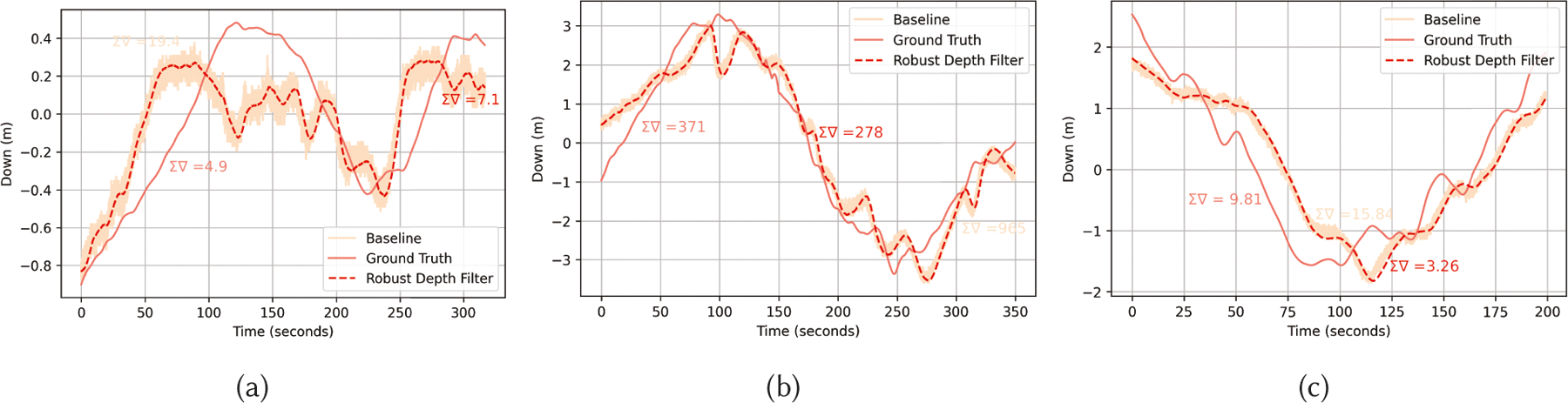
Sample z-axis trajectories of barometric g-h filter on three sequences in the AQUALOC dataset against baseline depth estimator. The sum of gradients for each plot is shown as well.

Fig. 10 shows sample trajectory reconstruction in the z-axis provided by the barometric *α* – *β* filter on the AQUALOC dataset. As a baseline, we convert the raw pressure sensor readings to depth using the formula *P*_*t*_ = *ρgL*_*z,t*_, where *P*_*t*_ is the raw pressure reading at time *t*, *ρ* is the density of seawater and *g* = 9.81. From Fig. 10, we can see that our filter is robust to sensor noise caused by environmental variations, with the sum of gradients of the reconstructed trajectory much closer to the ground truth trajectories. Compared to 3D-RoNIN and TLIO, which estimate height from IMU and have errors of up to ±1.0*m*, the barometric filter provides altitude estimates within ±0.1*m*. Moreover, 3D-RoNIN and TLIO use neural networks in the pipeline much larger than TinyOdom models to regress height. Our filter, on the other hand, is lightweight, requiring only 10 KB SRAM and 51.4 kB flash on the target hardware. In addition, our approach for regressing altitude is immune to the curse of drift associated with inertial sensors.

### 7.4 Ablation Study for Sequence Learning Formulation

**Fig. 11.**
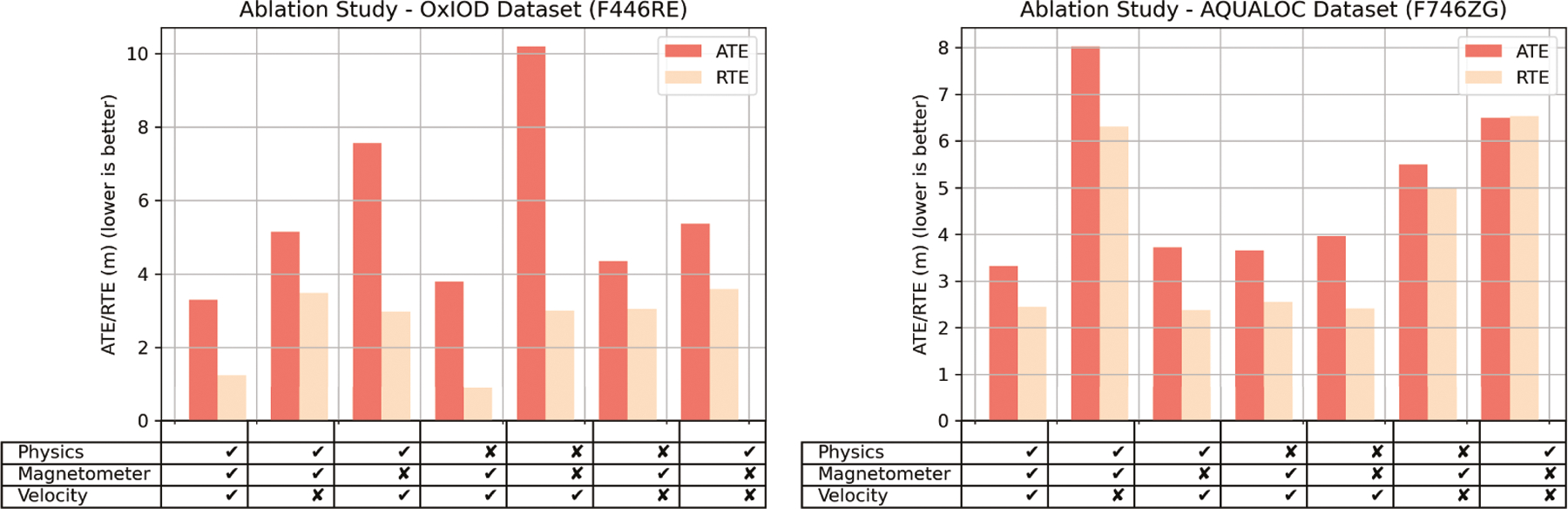
Ablation study showcasing the importance of velocity, magnetometer and physics-centric sequence learning formulation for models with same architectural encodings on the OxIOD and the AQUALOC dataset.

We performed an ablation study to highlight the importance of the individual components (i.e., velocity, magnetometer, and physics module) in our robust sequence learning formulation. We took a model, kept the same architectural encodings, and retrained the model without one or two of the three components. Fig. 11 summarizes the study performed on the OxIOD and AQUALOC dataset for the best performing models on STM32F446RE and STM32F746ZG hardware, respectively. From the ablation study, it is clear that the velocity formulation, magnetometer readings, and physics channels all work together to reduce the ATE and RTE of the same model. ATE and RTE are lowest when all three components are present in both datasets. In particular, the velocity-formulation and the inclusion of magnetometer seem to have the greatest effect in reducing the position estimation error. The velocity formulation solves the heading rate singularity issue associated with heading-displacement formulation, while the magnetometer provides an additional anchor for inferring body heading minus the effects of gravity pollution, varying sensor attitude, translational movements, and drift associated with accelerometers and gyroscopes. The physics channel, on the other hand, helps the neural network decide whether valid translational movements have occurred or not, thereby constraining the output of the network when the object to be localized is static.

### 7.5 Transferability and Real-World Evaluation

In this section, we first showcase how pre-trained models perform when they are tested on an entirely different dataset, which may or may not come from the same underlying application (Section 7.5.1). We then show how very small amounts of data in the new domain can fine-tune the weights of a pre-trained model trained on an entirely different underlying application and dataset to operate reasonably well in the real-world (Section 7.5.2).

#### 7.5.1 Transferability Across Applications and Datasets (No Fine-Tuning).

Table 6 provides an example of the performance of neural inertial models on new datasets and applications without fine-tuning. For testing the transferability across the same application but different datasets, we tested PDR models across the OxIOD and RoNIN datasets. For testing the transferability across different applications, we tested PDR (trained on the OxIOD dataset) models and UUV localization models (trained on the AQUALOC dataset). From Table 6, it is evident that while neural-inertial models (both TinyOdom and large models) perform well within the trained data distribution, they are not directly transferable to another dataset or application without fine-tuning. Apart from having different data distribution, these odometry datasets have different sampling rates as well as different motion primitives (e.g., a UUV’s motion patterns would be different from that of a person walking). Thereby, the ideal window sizes and learned physical embeddings would be significantly different across datasets and applications. In addition, the TinyOdom models perform slightly worse than the large models on different datasets and applications. This is because the lightweight models do not have enough redundant weights or parameters to model globally significant attributes that may be common across datasets or applications, but instead overfit the dataset-specific characteristics in the temporal sequences, sacrificing generalizability over accuracy. Thus, it is necessary to perform domain adaption when transferring pre-trained models to different applications.

#### 7.5.2 Fine-Tuning and Real-World Usage.

Before fine-tuning pre-trained models, we ran NAS using the entire 3-hour dataset to find TinyOdom models that can run in real-time on the target hardware listed in Table 3, as well as on the Razor IMU retrofitted to the robot. Table 7 lists the hardware and performance metrics of the best performing model for each hardware. Note that the ATE and RTE metrics shown for the Razor IMU are from the evaluation phase performed in real-time with the robot. From Table 7, we can observe that the RTE over 60 seconds is around 1 meter. This is acceptable for precision agriculture, as the robot can intermittently correct its location by fusing GPS with TinyOdom, while TinyOdom takes care of a high sampling rate and high-resolution cm-level localization.

**Fig. 12.**
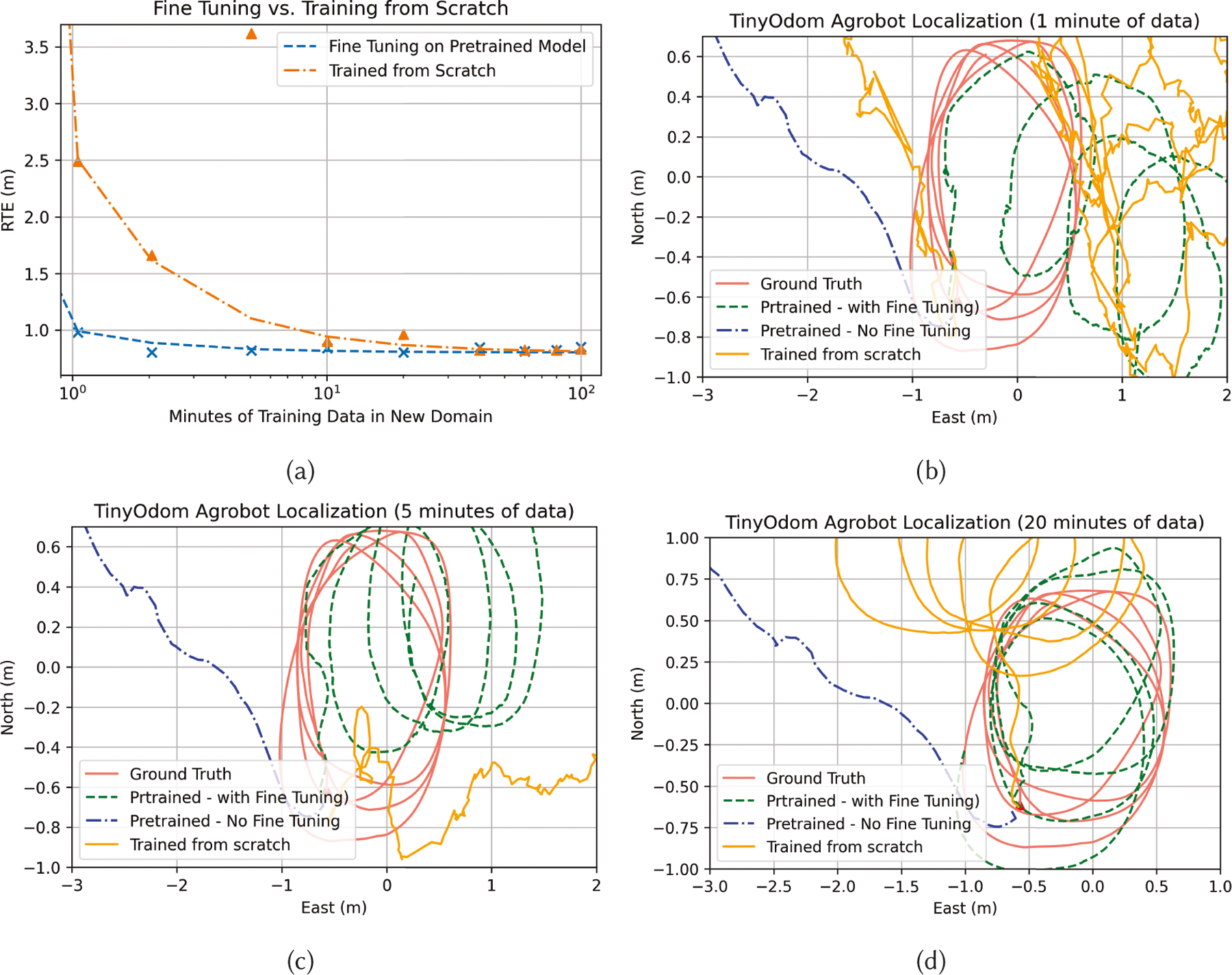
(a) Evolution of RTE with increase in availability of labelled data from new domain on pretrained TinyOdom model (pretrained on OxIOD) versus TinyOdom model trained from scratch. (b)(c) and (d) Trajectory reconstructions for Agrobot localization with a pretrained TinyOdom models (fine-tuned and not fine-tuned) and TinyOdom models trained from scratch. The minutes of data used by the pretrained TinyOdom model for fine-tuning and the model trained from scratch are shown.

Having empirical gurantees of TinyOdom capable of operating on real-world data in real-time on real hardware, next, we explored whether pretrained models trained on an entirely different application (hence dataset) can be fine tuned to operate in the real-world with limited labelled data from the new domain using transfer learning. Fig. 12(a) showcases how the localization error of a pretrained model (pretrained on the OxIOD dataset) evolve with the amount of labelled data in the new domain (agricultural robot positioning) available to the model. We compared the error evolution against a model trained from scratch using the same amount of limited data. We observed that the RTE of a pre-trained model with no fine-tuning reduces by 8× with only 1 minute of labeled data in the new domain. Moreover, from the graph, we can observe that a model being trained from scratch needs around 100 minutes of data in the new domain to match the RTE of the pre-trained model. Intuitively, pre-trained models bring in stability in error evolution which models trained from scratch cannot bring with limited data, even if the pre-trained model was trained on a different application. Fig. 12(b)(c)(d) shows trajectory reconstructions on unseen data for a 20m trajectory on 1 minute, 5 minutes, and 20 minutes of data in the new domain. The pre-trained model converges much faster to mimic the ground truth trajectory with an increase in available data, while the classifier trained from scratch struggles to converge with limited data.

## 8 CONCLUSION AND FUTURE WORK

Inertial odometry extends the applicability of odometry to GPS and network-denied environments. Although the conventional model-based inertial odometry approaches are computationally feasible, they demand expert heuristics impacting their generalizability. Further, the current data-driven approaches for inertial odometry models, at best, work on smartphone class devices and are not transferable to the URC devices. We show that the proposed TinyOdom framework introduces a robust 3D inertial sequence learning formulation and a hardwareaware NAS framework to train inertial odometry models. TinyOdom extends the neural-inertial odometry across heterogeneous applications while achieving up to 134× smaller size than the current SOTA while maintaining or exceeding localization resolution. Our evaluation shows that the performance of existing data-driven inertial odometry is susceptible to the vanilla sequence learning formulation. The widely adopted heading displacement-based formulation is adversely impacted by heading-rate singularity (e.g., during share turns in the trajectory). In contrast, the introduced magnetometer, physics, and velocity-centric inertial sequence formulation of TinyOdom maintain or exceed the tracking performance over the SOTA, even though the model sizes are significantly smaller. Finally, we showcased the challenges that arise when trying to transfer pre-trained neural-inertial models in the real world and showcased transfer learning as a potential solution. There are several avenues of future work. *Firstly*, we observed that in the absence of GPS or infrastructure-provided location fixes, the errors in TinyOdom and other SOTA inertial odometry approaches are cumulative with time. This suggests for long-term usage, inertial odometry necessitates intermittent error correction from the infrastructure within the available resource budget. *Secondly*, we saw that complementing neural-inertial models with heuristics and physics improves the robustness dramatically. Our physics metadata module is not trainable so far and uses domain knowledge. Possible use of neurosymbolic reasoning [92], physics-aware embeddings [21][95], or signal temporal logic [60] may help improve neural network robustness without domain expertise. *Thirdly*, neural-inertial models trained on one dataset do not directly transfer to another dataset even when the application is the same, let alone different applications. While our transfer learning approach with a few minutes of labeled data fine-tunes pre-trained models for real-world usage, more work needs to be done for on-device fine-tuning [26] and possible use of unsupervised pre-trained embeddings [7]. *Finally*, the resolution achieved by neural-inertial odometry models depends on the best possible resolution of the ground truth hardware. However, developers may not have access to high-resolution sub-mm accuracy motion capture systems, particularly when operating over a large geographical region. Therefore, efficient ways to log high-resolution ground-truth data need to be explored.

## Figures and Tables

**Table 1.** List of datasets used for evaluation. We use five datasets spanning four different applications.

Application	Dataset	Environment	Device Configuration	Device Placement	Ground Truth	Data Specifications
Pedestrian Dead Reckoning	OxIOD [18]	Indoors	Smartphone 9DoF-IMU (InvenSense 2600)	Hand, pocket, bag, trolley	Vicon	5 subjects, 14.7 hours, 42.6 km
	RoNIN* [46]	Indoors	Smartphone 9DoF-IMU (ICM20602, LSM6DSL)	Unrestricted	Tango	100 subjects, 42.7 hours
UUV Localization	AQUALOC [31]	Underwater	9DoF-IMU (MPU-9250), Pressure Sensor (MS5837–30BA, Keller 7LD-100BA)	Fixed, underwater probe	ColMap	2 probes, 1.74 hours, 0.78 km
UAV Localization	EuRoC MAV [13]	Indoors	6DoF-IMU (ADIS 16488)	Fixed, MAV	Vicon	1 MAV, 0.37 hours, 0.9 km
Animal Tracking	GunDog [41]	Outdoors	6DoF-IMU (no gyroscope)	Fixed, penguin	GPS	1 animal, 7.6 km

* only 50% of the dataset is publicly available.

**Table 2.** Window size, stride,training-validation-test splits (by sequences), and training epochs used in the benchmark datasets. The validation split is used to compute error metric during NAS and not for individual model training. The test split is used for final evaluation of the model with most optimal hyperparameters found via NAS.

Dataset	Sampling Rate (Hz)	Window Size	Stride	Splits (Tr, Val, Te) (%)	Model Epochs	NAS epochs
OxIOD	100	200	10	85, 5, 10	900	50
RoNIN	200	400	20	70, 5, 25	900	50
AQUALOC	200	400	20	80, 5, 15	300	30
EuRoC MAV	200	50	5	80, 10, 10	300	30
GunDog	40	10	10	45*, 5*, 50	300	30

* Training trajectory split into 2 parts for train and validation splits.

**Table 3.** List of hardware evaluated for NAS.

Hardware	SRAM (kB)	Flash (kB)	Proxy/HIL
STM32F446RE	128	512	HIL, Proxy
STM32L476RG	128	1024	HIL, Proxy
STM32F407VET6	192	512	Proxy
STM32F746ZG	320	1024	HIL, Proxy

**Table 4.** GPU workstations used to perform NAS.

Processor	GPU	RAM (GB)
3.7 GHz AMD Ryzen Threadripper 3970x 32 core	2× 24 GB Nvidia GeForce RTX 3090	256
2.5 GHZ Intel Xeon W-2175 14 core	2× 24 GB Nvidia Titan RTX	256
3.4 GHz AMD Ryzen Threadripper 1950X 16 core	2× 12 GB Nvidia GeForce GTX 1080 Ti	128
3.0 GHz Intel Core i7–6050x 10 core	2× 12 GB Nvidia GeForce GTX Titan X	128
3.4 GHz Intel Core i7–2600k 4 core	1× 12 GB Nvidia GeForce GTX Titan X	32

**Table 5.** Performance of competing 2D inertial dead-reckoning techniques for human, UUV, UAV and animal tracking against TinyOdom in terms of ATE and RTE (lower is better). The top three techniques are shaded. For TinyOdom models, the corresponding hardware are shown in parenthesis.

Application	Dataset	Method	SRAM (kB)	Flash (kB)	FLOPS (M)	ATE (m)	RTE (m)
Pedestrian Dead Reckoning	OxIOD [18]	PDR [48]	10.8	49.6	-	3.47	3.24
		NDI [88]	1.2	28.1	-	9119.50	247.53
		IONet [16]	766.7	679.5	-	5.95	2.84
		L-IONet [18]	154.2	182.9	13.87	4.37	2.82
		**RoNIN TCN** [46]	2046.3	**2195.5**	**220**	**1.95**	**0.42**
		TinyOdom (STM32F446RE)	52.4	71.6	4.64	3.30	1.24
		TinyOdom (STM32L476RG)	72.5	89.6	6.65	3.59	1.37
		TinyOdom (STM32F407VET6)	90.1	117.6	8.92	6.82	1.28
		TinyOdom (STM32F746ZG)	55.5	71.0	4.92	2.80	1.26
	RoNIN [46]	PDR [48]	10.8	49.6	-	34.81	23.62
		NDI [88]	1.2	28.1	-	12398.00	59.85
		IONet [16]	976.3	782.0	-	22.52	7.63
		L-IONet [18]	159.0	182.9	26.8	24.73	14.84
		**RoNIN TCN** # [46]	2046.3	**2195.5**	**440**	**4.73**	**1.21**
		TinyOdom (STM32F446RE)	36.2	50.8	4.15	28.3	7.76
		TinyOdom (STM32L476RG)	56.2	65.3	7.80	23.9	6.74
		TinyOdom (STM32F407VET6)	138.3	147.1	26.54	27.7	6.20
		TinyOdom (STM32F746ZG)	257.3	253.9	49.44	27.36	5.84

UUV Localization	AQUALOC [31]	NavNet [96]	1364.1	1396.5	-	3.80	2.98
		TinyOdom (STM32F446RE)	18.4	20.5	0.34	4.99	2.78
		TinyOdom (STM32L476RG)	36.7	34.2	4.03	4.90	3.30
		TinyOdom (STM32F407VET6)	18.6	20.9	0.40	4.03	2.83
		**TinyOdom (STM32F746ZG)**	**17.3**	**26.8**	**0.14**	**3.32**	**2.45**

UAV Localization	EuRoC MAV [13]	AbolDeepIO [29]	4217.9	4217.8	-	11.24	13.96
		VeTorch [35]	7325.3	7294.1	899.74	13.5	15.2
		TinyOdom (STM32F446RE)	87.5	110.0	2.95	2.82	2.41
		TinyOdom (STM32L476RG)	89.8	110.2	3.18	2.24	2.25
		TinyOdom (STM32F407VET6)	63.8	85.6	2.14	2.90	2.55
		**TinyOdom (STM32F746ZG)**	**4.37**	**31.4**	**0.073**	**2.19**	**2.02**

Animal Tracking	GunDog [41]	GunDog [41, 88]	8.5	32.5	-	28.45	10.53
		TinyOdom (STM32F446RE)	49.9	72.8	2.92	43.9	0.27
		TinyOdom (STM32L476RG)	32.4	55.9	1.81	60.82	0.35
		TinyOdom (STM32F407VET6)	84.3	95.9	3.19	28.2	0.20
		**TinyOdom (STM32F746ZG)**	**45.4**	**64.0**	**1.90**	**11.41**	**0.15**

# trained on entire RoNIN dataset, while only 50% is publicly available [46].

FLOPS calculation for models with LSTM/GRU/RNN cells were inaccurate and hence ommitted.

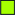
 and bolded - best performing technique, 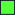
- second best performing technique, 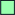
- third best performing technique.

**Table 6.** RTE (m) of neural-inertial models across different datasets (left) and applications (right) without fine tuning. The dataset on which the model is trained on is shown in parenthesis.

Method (Training Dataset)	OxIOD	RoNIN
IONet (OxIOD) IONet (RoNIN)	2.84 7.65	4.7 7.63
LIONet (OxIOD) LIONet (RoNIN)	2.82 8.35	4.7 14.84
RoNIN TCN (OxIOD) RoNIN TCN (RoNIN)	0.42 10.3	13.4 1.21
TINYODOM (OxIOD) TINYODOM (RoNIN)	1.26 3.16	97.2 6.74

Method (Trained Application)	PDR	UUV
IONet (PDR)	2.84	5.15
LIONet (PDR)	2.82	3.94
RoNIN TCN (PDR)	0.42	14.96
TinyOdom (PDR)	1.26	7.83
NavNet (UUV)	93	2.96
TINYODOM (UUV)	5.82	2.45

**Table 7.** Resource and localization metrics for TinyOdom models geared towards different hardware for agricultural robot localization trained using the entire dataset (3 hours) from scratch. The ATE is for a 20 minute (250 m) trajectory.

Hardware	SRAM (kB)	Flash (kB)	FLOPS (M)	ATE (m)	RTE (m)
Razor IMU	3.1	16.2	0.012	15.3	1.13
STM32F446RE	4.1	32.4	0.059	24.5	1.47
STM32L476RG	4.6	17.8	0.062	18.3	1.02
STM32F407VET6	15.6	31.9	0.359	22.96	1.28
STM32F746ZG	3.2	19.8	0.016	19.65	0.96

## CCS Concepts:

**• Computing methodologies → Machine learning**; **• Computer systems organization →** *Robotics*; Embedded systems.

## Footnotes

1
https://github.com/nesl/tinyodom

2
https://lopsi.weebly.com/teaching.html

3
https://github.com/Sachini/ronin

4
https://github.com/Richard6195/Dead-reckoning-animal-movements-in-R

5
https://optitrack.com/cameras/prime-17w/

6
https://optitrack.com/software/motive/
